# An Innovative Approach to Precisely Tailor the Composition of Syngas in CO_2_ Electroreduction

**DOI:** 10.1002/advs.202505424

**Published:** 2025-06-23

**Authors:** Shuai Lv, Xinyi Sun, Baolin Wang, Wenrui Wan, Li Wang, Jianji Wang, Jinglai Zhang

**Affiliations:** ^1^ Henan Key Laboratory of Protection and Safety Energy Storage of Light Metal Materials College of Chemistry and Molecular Sciences Henan University Kaifeng Henan 475004 P. R. China; ^2^ Key Laboratory of Green Chemical Media and Reactions (Ministry of Education) Collaborative Innovation Centre of Henan Province for Green Manufacturing of Fine Chemicals School of Chemistry and Chemical Engineering Henan Normal University Xinxiang Henan 453007 P. R. China

**Keywords:** **CuIn/BP** electrocatalyst, electrocatalytic reduction of CO_2_, linear relationship, precise regulation, syngas

## Abstract

Syngas production through CO_2_ electrochemical reduction reaction (CO_2_RR) has attracted numerous attention for reducing carbon emissions and increasing chemical feedstocks. However, the precise regulation of H_2_/CO molar ratio in syngas remains a challenge, especially through simple and feasible methodologies. In this work, a novel composite electrocatalyst, copper‐indium/black phosphorus (**CuIn/BP**) is developed, using a simple one‐step co‐reduction method for efficient CO_2_RR. It is found for the first time that there is a linear relationship between BP content in **CuIn/BP** and the H_2_/CO molar ratio in syngas. Consequently, the H_2_/CO molar ratio in syngas can be precisely tailored by simply adjusting the BP content in **CuIn/BP**. A total faradaic efficiency (FE) of CO and H_2_ reached ≈ 100% along with a current density of 135.2 mA cm^−2^. Mechanistic studies suggest that the CuIn phase and Cu_3_P species are in situ generated in **CuIn/BP**, where the CuIn phases mainly contribute to CO generation, while the Cu_3_P species promote H_2_ formation. The variation in BP content modulates the proportion of the CuIn phase and Cu_3_P species, leading to the variations in H_2_/CO molar ratios. Thus, this study provides an efficient and simple strategy to tailor the H_2_/CO molar ratio in syngas for various industrial applications.

## Graphical abstract

A new **CuIn/BP** (copper‐indium/black phosphorus) heterostructure catalyst is designed and synthesized by simple one‐step co‐reduction. A linear correlation is observed between the H_2_/CO molar ratio and the content of BP in CuIn/BP for the first time. By simply altering the content of BP in the catalyst, H_2_/CO ratio in syngas could be precisely tuned on demand for downstream industrial processes.

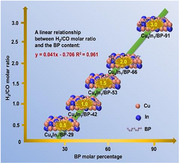

## 1 Introduction

Over the past century, the massive consumption of fossil fuels has led to a continuous increase in carbon dioxide (CO_2_) concentration in the atmosphere, thereby triggering severe climate issues and environmental problems.^[^ ^1^, ^2^ ^]^ Electrochemical reduction of CO_2_ driven by renewable electricity is regarded as a promising solution to reduce CO_2_ emissions and lessen dependence on traditional fossil resources.^[^ ^3^, ^4^, ^5^ ^]^ Among the various reduction products, syngas, a mixture of hydrogen (H_2_) and carbon monoxide (CO), has garnered widespread attention due to its crucial role in the Fischer‐Tropsch process, where the ultimate product largely depends on the H_2_/CO molar ratio.^[^ ^6^, ^7^ ^]^ To date, extensive efforts have been devoted to refining the activity and selectivity of electrocatalysts to achieve a controllable H_2_/CO ratio, and significant progress has been made in this area.^[^ ^8^, ^9^, ^10^ ^]^

Monometallic electrocatalysts are popular and common candidates for CO_2_ reduction reaction (CO_2_RR) to produce CO or syngas, such as palladium (Pd), gold (Au), and silver (Ag).^[^ ^11^, ^12^, ^13^ ^]^ Typically, they modulate the molar ratio of H_2_ to CO in syngas by promoting the generation of CO and suppressing the hydrogen evolution reaction (HER) which is the inevitable competing reaction of CO_2_RR in aqueous media. The capacity of a single metal center is constrained in simultaneously regulating the HER and CO_2_RR.^[^ ^14^ ^]^ Therefore, it is challenging to produce syngas with the desired H_2_/CO ratio based on monometallic electrocatalysts. This limitation has led researchers to explore more robust catalyst systems, and therefore, the introduction of additional active sites has spurred the advancement of bimetallic electrocatalysts,^[^ ^15^ ^]^ where two distinct active sites are employed to promote HER and CO_2_RR, respectively.^[^ ^16^ ^]^ For example, Du et al. utilized the zinc‐lanthanum (Zn‐La) dual atomic catalysts to produce syngas achieving CO/H_2_ molar ratios of 1 and 0.5 by adjusting the relative amount of Zn and La, where the Zn and La centers are responsible for the production of CO and H_2_, respectively.^[^ ^17^ ^]^ Alternatively, different metallic active sites are employed to regulate the generation of CO by modulating the different electron transfer processes in CO_2_RR, such as the adsorption of *COOH and desorption of *CO, to achieve the ultimate goal of controlling the H_2_/CO molar ratio.^[^ ^18^ ^]^ The CO/H_2_ molar ratio ranging from 0.25 to 2.50 over a narrow potential window (−0.7 to −1.1 V versus the reversible hydrogen electrode (versus RHE)) is regulated by bimetallic AuZn/ZnO catalyst due to the different adsorption abilities of the metallic active species toward *COOH and *CO.^[^ ^19^ ^]^ In addition to the composition, microstructure, phase, and exposed crystal plane also significantly influence catalytic behavior.^[^ ^20^, ^21^, ^22^ ^]^ However, synthesizing bimetallic catalysts with various arrangements is significantly more challenging compared to modulating composition. Although a wide range of H_2_/CO ratios ranging from 0.1 to 25 has been achieved in various systems,^[^ ^8^, ^18^, ^19^ ^]^ none could reliably tailor specific ratios through deliberate catalyst composition modulation.

It is reported that Cu‐based monometallic electrocatalysts are able to reduce CO_2_ to hydrocarbon products due to their weak *H binding and strong *CO binding ability.^[^ ^23^ ^]^ When a small amount of In_2_O_3_ is introduced to form Cu/In_2_O_3_, the CO selectivity is greatly improved to 95%,^[^ ^24^ ^]^ since the existence of In_2_O_3_ prevents the oxidation of Cu and alters its electronic structure. Thus, it is inferred that the **CuIn** bimetallic catalyst may display excellent selectivity for CO generation. On the other hand, black phosphorus (BP) nanosheets exhibit promising potential in water‐splitting reactions to generate H_2_ because of their weak adsorption energy for *H and strong adsorption energy for *O‐containing intermediates.^[^ ^25^, ^26^ ^]^ The incorporation of BP into **CuIn** is likely to promote H_2_ production. Therefore, by integrating **CuIn** with BP to form a **CuIn/BP** electrocatalyst, it is possible to finely tune the H_2_/CO molar ratio.

In this work, a novel electrocatalyst, **CuIn/BP**, was synthesized through a straightforward one‐step co‐reduction method, where BP was introduced into **CuIn** nanoparticles to form a heterostructure. It was found that when using **CuIn/BP** as an electrocatalyst, the current density reached 135.2 mA cm^−2^ in a flow cell at −0.6 V versus RHE, and the H_2_/CO molar ratio could be tuned from 0.03 to 5.0 within a potential window of −0.5 to −0.9 V versus RHE. Interestingly, a linear relationship was observed between the H_2_/CO molar ratio and the BP content in **CuIn/BP**. It was feasible to obtain any specific H_2_/CO ratio suitable for downstream industrial processes by merely varying the BP content in **CuIn/BP**. The In metal incorporated in **CuIn/BP** stabilized the Cu^+^ species, which played a major role in assisting CO formation. At the same time, the in situ‐generated CuIn phase promotes CO production, while in situ‐generated Cu_3_P species enhances HER, as evidenced by in situ attenuated total reflectance Fourier transform infrared spectroscopy (ATR‐FTIR), high‐resolution transmission electron microscopy (HRTEM), X‐ray photoelectron spectroscopy (XPS), and density functional theory (DFT) calculations.

## 2 Results and Discussion

### 2.1 Catalysts Characterization

**Figure 1:**
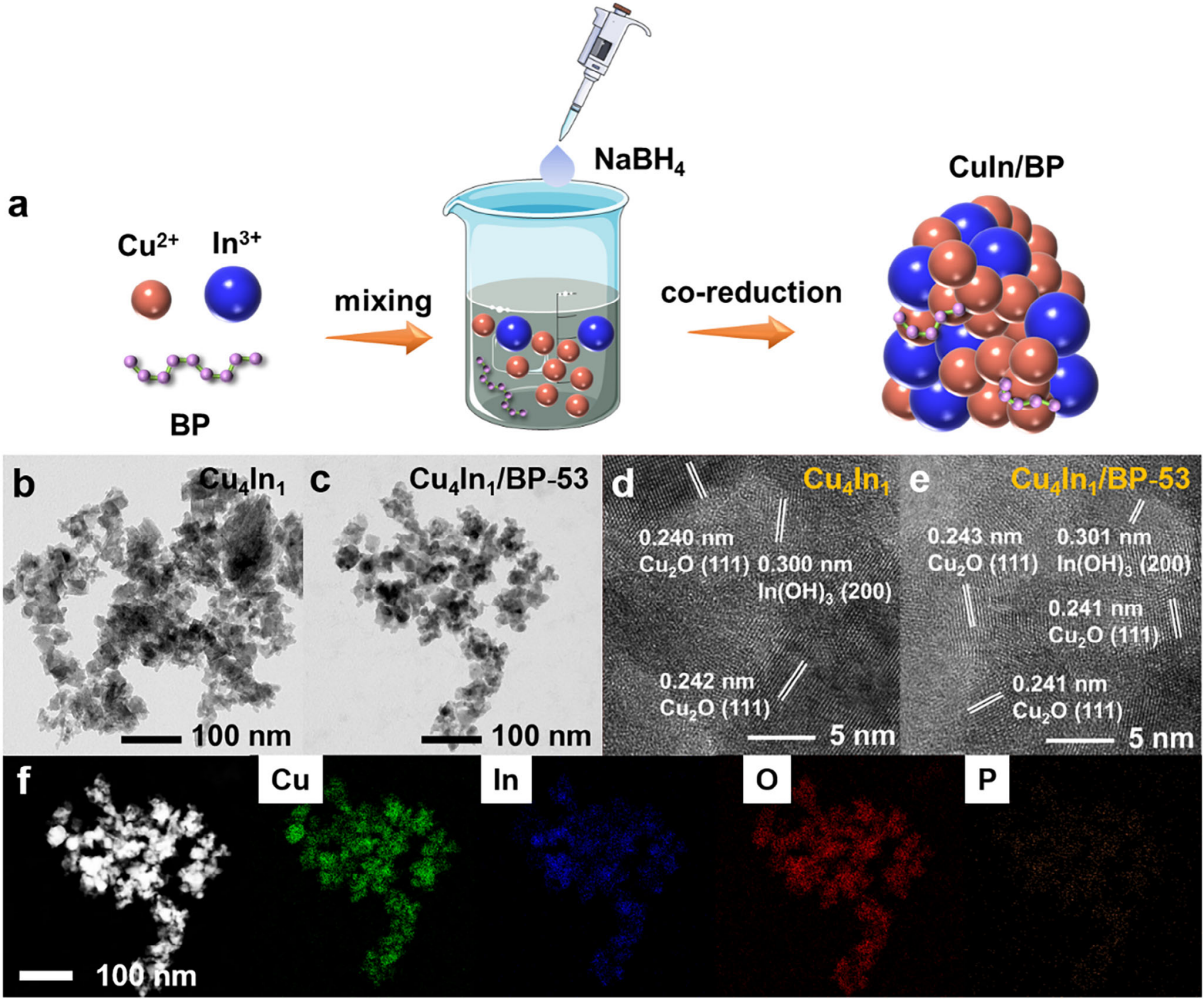
(a) The synthetic scheme of **CuIn/BP**. TEM images of (b) Cu_4_In_1_ and (c) Cu_4_In_1_/BP‐53. HRTEM images of (d) Cu_4_In_1_ and (e) Cu_4_In_1_/BP‐53. (f) EDS elemental mapping of Cu_4_In_1_/BP‐53. Note that the subscripts 4 and 1 represent the molar ratio of Cu to In in Cu_4_In_1_ and Cu_4_In_1_/BP‐53, and the total molar mass of Cu and In is 2 mmol, while 53 indicates that the molar percentage of BP in Cu_4_In_1_ is 0.53. If no special indication is given, the numbers in other samples also represent the same meaning.

**CuIn** and **CuIn/BP** samples, denoted as Cu_4_In_1_ and Cu_4_In_1_/BP‐53, were synthesized via a one‐step co‐reduction method, as schematically shown in **Figure** **1**a. Here, the subscripts 4 and 1 represent the molar ratio of Cu to In in Cu_4_In_1_ and Cu_4_In_1_/BP‐53, and the total molar mass of Cu and In fixed at 2 mmol, while 53 indicates that the molar percentage of BP in Cu_4_In_1_ is 0.53, and the same meaning applies in the next discussions. The morphology of the samples was characterized by scanning electron microscopy (SEM) (Figure S1a,b, Supporting Information) and transmission electron microscopy (TEM) (Figure 1b,c). Both samples are composed of irregularly shaped particles with an average particle size of 30 nm, and no significant difference in morphology was observed between them implying the negligible influence of BP doping on the morphology. Examining the high‐resolution TEM (HRTEM) image of Cu_4_In_1_ (Figure 1d), lattice spacings of 0.240, 0.242, and 0.300 nm were observed, which are in good agreement with the Cu_2_O (111) and In(OH)_3_ (200) planes.^[^ ^27^ ^]^ Considering the standard reduction potentials in an aqueous solution,^[^ ^28^ ^]^ copper and indium ions can be reduced to their metallic form by NaBH_4_. However, NaBH_4_ typically undergoes hydrolysis in an aqueous solution, leading to a gradual loss of its reducing power.^[^ ^29^ ^]^ The presence of Cu_2_O may be ascribed to the insufficient reduction of Cu^2+^ ions, and In(OH)_3_ is generated as a by‐product in the aqueous solution due to the faster hydrolysis of NaBH_4_ compared to In^3+^ reduction. In the HRTEM image of Cu_4_In_1_/BP‐53 (Figure 1e), the aforementioned lattice spacings still existed except for slight variation to 0.243 nm (Cu_2_O (111)), 0.241 nm (Cu_2_O (111)) and 0.301 nm (In(OH)_3_ (200)), respectively. The difference in lattice spacing variation may be attributed to the differing interactions between Cu and In atoms in Cu_4_In_1_ and Cu_4_In_1_/BP‐53.^[^ ^30^ ^]^ As control samples, pristine **Cu** and **In** samples were also synthesized by reduction of Cu^2+^ and In^3+^ ions with NaBH_4_, respectively. The TEM images of pristine **Cu** and **In** (Figure S2a,c, Supporting Information) revealed that both samples are also composed of irregularly shaped particles. HRTEM analysis (Figure S2b,d, Supporting Information) further clarifies that the dominant crystallographic plane is Cu_2_O (111) and In(OH)_3_ (200). Notably, the presence of Cu (111) plane in the **Cu** sample and the In (101) plane in the **In** sample suggests that Cu^2+^ and In^3+^ have undergone partial reduction, but these features are absent in the Cu_4_In_1_ and Cu_4_In_1_/BP‐53 samples, suggesting that the synergistic effect between Cu and In played a decisive role in the formation of the reduction products. Based on the energy dispersive X‐ray spectroscopy (EDS) mapping result (Figure S3, Supporting Information; Figure 1f), the Cu, In, and O elements are uniformly dispersed in the Cu_4_In_1_ sample, and Cu, In, O, and P elements are also evenly distributed in Cu_4_In_1_/BP‐53 sample. The EDS mapping revealed that the molar ratio of Cu to In in Cu_4_In_1_/BP‐53 and Cu_4_In_1_ are 3.3:1 and 2.7:1, respectively (Table S2, Supporting Information), which is in line with the results from inductively coupled plasma optical emission spectrometry (ICP‐OES), where the molar ratio of Cu and In in Cu_4_In_1_/BP‐53 and Cu_4_In_1_ was 3.2:1 and 3.0:1, respectively.

**Figure 2:**
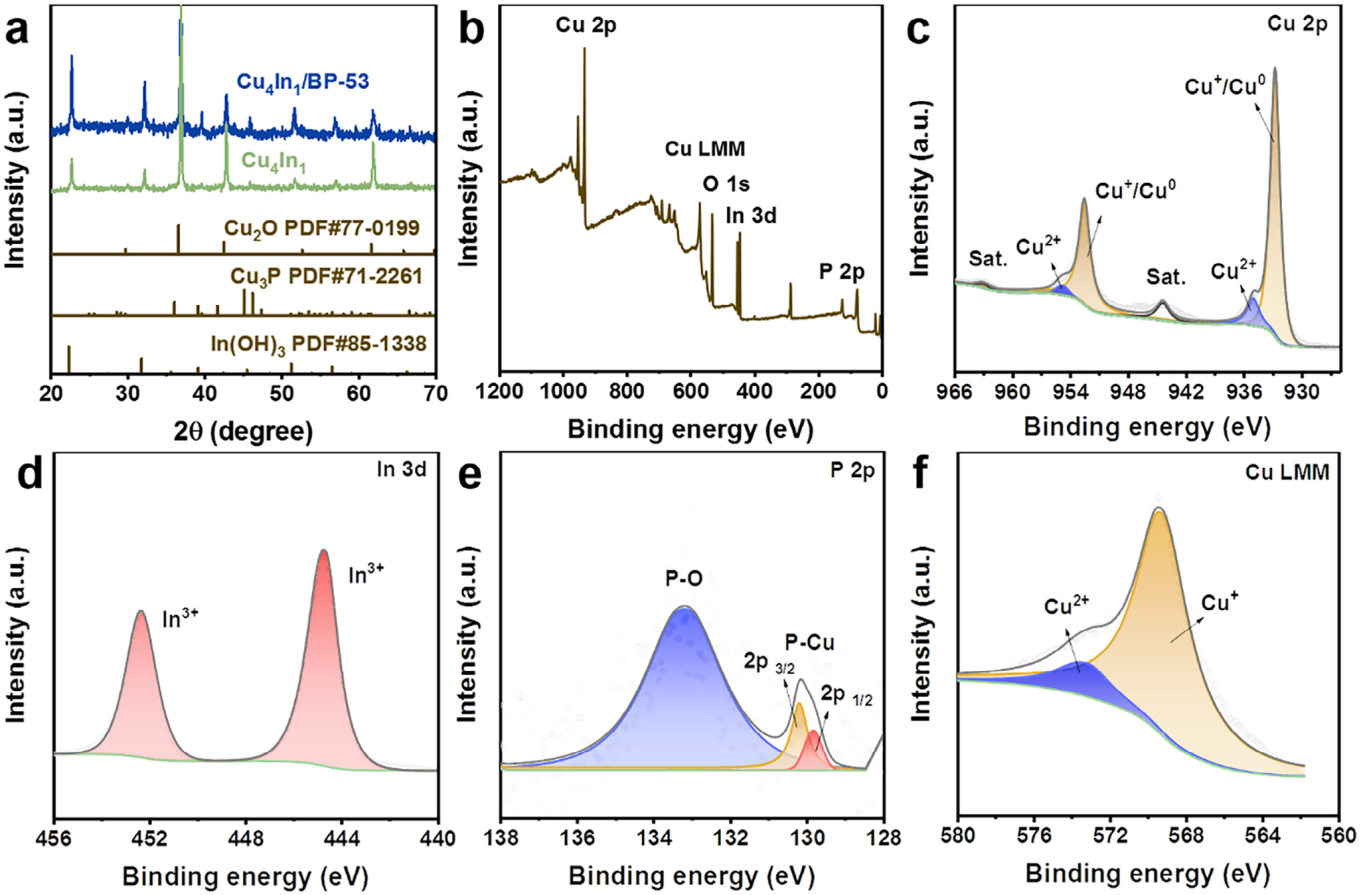
(a) XRD patterns of Cu_4_In_1_/BP‐53 and Cu_4_In_1_, (b) Full XPS survey spectrum of Cu_4_In_1_/BP‐53, and high‐resolution XPS spectra of (c) Cu 2p, (d) In 3d, (e) P 2p, and (f) Cu LMM.

The crystal structures of Cu_4_In_1_/BP‐53 and Cu_4_In_1_ were analyzed by X‐ray diffraction (XRD) patterns as shown in **Figure** **2**a. For comparison, the XRD patterns of pristine **Cu** and **In** samples were also determined and presented in Figure S4 (Supporting Information). Unlike the pristine **Cu** and **In** samples which contain metallic phases of Cu and In, respectively, only the Cu_2_O phase, with diffraction peaks at ≈32.5°, ≈42.4° and ≈61.6° and the In(OH)_3_ phase with diffraction peaks at ≈22.3°, ≈31.7°, ≈51.2° and ≈56.5°, were present in the Cu_4_In_1_ and Cu_4_In_1_/BP‐53 samples. These results are in agreement with the HRTEM analysis. Additionally, the distinctive peaks at ≈39.1° and ≈45.1° are indicative of Cu_3_P formation implying that a heterostructure between Cu_4_In_1_ and BP, rather than a physical mixture is formed in the electrocatalyst.^[^ ^31^, ^32^ ^]^

XPS measurements were performed to investigate valence states and the components of Cu_4_In_1_/BP‐53 sample. The XPS results for pristine **Cu**, **In**, and Cu_4_In_1_ samples are displayed in Figures S5 and S6 (Supporting Information), respectively. In the full survey spectrum (Figure 2b), the sample consists of Cu, In, O, and P elements, confirming the EDS mapping results. In the Cu 2p high‐resolution spectrum (Figure 2c), the binding energy peaks at 932.6 and 952.1 eV are assigned to Cu^+^/Cu^0^ species. The peaks at 933.7 and 954.0 eV are indicative of Cu^2+^, and the existence of satellite peaks at 943.0 and 962.6 eV confirm copper oxidation due to exposure to air.^[^ ^33^ ^]^ In the In 3d high‐resolution spectrum (Figure 2d), the peaks located at 444.9 and 452.5 eV are assigned to In^3+^ ions, suggesting that In is present as In(OH)_3_ within the Cu_4_In_1_/BP‐53 sample, and this conclusion is supported by the integrated analysis of XRD and HRTEM results.^[^ ^34^ ^]^ The P 2p high‐resolution spectrum (Figure 2e) shows a peak at 132.8 eV originating from the P‐O bond indicating the existence of oxidized phosphorus species. The peaks at 129.1 and 130.2 eV correspond to the binding energies of P 2p_3/2_ and P 2p_1/2_, respectively, and the determination of Cu‐P bond in the P 2p spectrum further demonstrates the formation of copper phosphide, which should be Cu_3_P according to the above XRD result.^[^ ^35^ ^]^ Furthermore, considering the close binding energies of Cu^+^ and Cu^0^ in the Cu 2p spectrum, a comprehensive investigation of the surface state was carried out using a Cu LMM Auger spectroscopy (Figure 2f). The Cu LMM analysis clearly reveals that Cu^+^ is present in the Cu_4_In_1_/BP‐53 sample except for Cu^2+^ results from oxidation. Furthermore, Cu LMM analysis indicates that the Cu^+^ content in the **Cu** sample (Figure S5, Supporting Information) accounts for 62.3% of the total Cu content. In Cu_4_In_1_ (Figure S6, Supporting Information) and Cu_4_In_1_/BP‐53 samples, the Cu^+^ content increases to 83.6% and 85.7%, respectively. This increase indicates that there is an interaction between Cu and In as well as between Cu and BP.

### 2.2 Electrochemical Test for Cu_4_In_1_

To examine the catalytic activity of different samples for electrochemical CO_2_ reduction, linear scanning voltammetry (LSV) curves were collected in a N_2_/CO_2_‐saturated aqueous 0.5 M KHCO_3_ electrolyte (Figure S7, Supporting Information). It is clear that almost no reaction is observed in either N_2_‐saturated or CO_2_‐saturated electrolyte for the **In** sample (Figure S7a, Supporting Information) as In(OH)_3_ does not exhibit any activity for CO_2_.^[^ ^36^ ^]^ In Figure S7b,c (Supporting Information), **Cu** and Cu_4_In_1_ samples exhibit catalytic activity for CO_2_RR, as evidenced by the more positive potential and the higher current density in the CO_2_‐saturated electrolyte relative to that in the N_2_‐saturated electrolyte. The faradaic efficiency (FE) was further evaluated by potentiometric measurements to quantitatively compare the catalytic selectivity of **Cu** and Cu_4_In_1_. The gas‐phase products (CO and H_2_) were analyzed using gas chromatography (GC, Panna A91 Plus) and are shown in Figure S8 (Supporting Information), while the liquid‐phase products, primarily formic acid (HCOOH), were measured by ^1^H nuclear magnetic resonance (^1^H NMR, Bruker Plus 600 MHz) spectroscopy (Figure S9, Supporting Information). As shown in Figure S10 (Supporting Information), the maximum FE_CO_ over **Cu** is only 37.4% at −0.5 V versus RHE, while it increases to 96.7% for Cu_4_In_1_, indicating the necessity of In species to improve the selectivity toward CO production.

Additionally, the LSV curves were also collected for Cu_1_In_1_, Cu_8_In_1_, and Cu_16_In_1_ in a CO_2_‐saturated 0.5 M KHCO_3_ electrolyte to determine the optimal molar ratio between Cu and In in **CuIn** samples. As displayed in Figure S11a (Supporting Information), the current density basically increases with the increment of Cu content in the **CuIn** sample to achieve a maximum when the molar ratio of Cu to In is 4:1. However, the current density slightly reduces with the further increased Cu component. In general, the Cu content in **CuIn** plays a critical role in promoting the CO_2_RR to CO. Subsequently**, t**he **CuIn** samples for CO_2_RR were tested at five different applied potentials. As displayed in Figure S11b–f (Supporting Information), the products for CO_2_RR over Cu_1_In_1_ include CO, HCOOH, and H_2_ with the FE_HCOOH_ reaching 27.2%. Notably, the FE_HCOOH_ declines with the increasing Cu content in **CuIn** and reaches zero when the molar ratio of Cu to In is over 4:1. In this case, the sum of FE_CO_ and $FE_{H_{2}}$ is close to 100% for Cu_4_In_1_, Cu_8_In_1_, and Cu_16_In_1_ meaning that the HCOOH production is completely suppressed. For Cu_4_In_1_, the FE_CO_ gradually decreases from 96.5% at −0.6 V versus RHE to 68.8% at −0.9 V versus RHE with the increased potential due to the significant hydrogen evolution. A similar behavior is observed for Cu_8_In_1_ and Cu_16_In_1_, however, the maximum FE_CO_ reduces to 93.6% and 87.5% at −0.6 V versus RHE for the latter two samples, respectively. Overall, Cu_4_In_1_ is the most suitable candidate for CO production.

To gain insights into the critical items contributing to the superior activity of the Cu_4_In_1_ sample, XPS spectroscopy and HRTEM analysis on Cu_1_In_1_ and Cu_16_In_1_ samples were performed and compared. The high‐resolution XPS spectra, as shown in Figure S12a,b (Supporting Information), reveal discrepancies in both the peak intensities and positions of copper and indium across the three samples. In all samples, only In^3+^ is determined in the In 3d high‐resolution spectrum, which is attributed to In(OH)_3_, and the concentration of In^3+^ diminishes progressively with increasing Cu content in the **CuIn** samples. In the **CuIn** samples, Cu^+^ or Cu^0^ is predominant, and the concentrations of Cu^2+^ increase with increasing Cu content. The Cu LMM Auger spectra for Cu_1_In_1_ and Cu_16_In_1_ samples, depicted in Figure S13 (Supporting Information), further disclose the valence state of Cu species. No metallic Cu was detected in the Cu_1_In_1_ sample, similar to the Cu_4_In_1_ sample. Conversely, a small amount of metallic Cu is detected in the Cu_16_In_1_ sample since the lower In content was insufficient to stabilize Cu^+^, leading to the reduction of a small amount of Cu^2+^ to metallic copper. The Cu^+^ content in the Cu_1_In_1_ and Cu_16_In_1_ samples is 80.5% and 69.0%, respectively, both lower than that in Cu_4_In_1_ (83.6%). The higher Cu^+^ content results in the superior catalytic performance of Cu_4_In_1_ since the Cu^+^ plays a critical role in CO_2_RR. Moreover, HRTEM analysis (Figure S14, Supporting Information) confirms the valence states of Cu and In in Cu_1_In_1_ and Cu_16_In_1_ samples, consistent with the XPS results. The shifts in peak positions observed in the XPS spectra along with the stretching and contraction of lattice fringes in the HRTEM images are attributed to the differing interactions between Cu and In in the **CuIn** samples.

The above results clearly indicate that an interaction between Cu and In species in Cu_4_In_1_, but this interaction is not present in the fresh Cu_4_In_1_. Thus, it is likely that a new phase is generated in situ during CO_2_RR. To verify this, the Cu_4_In_1_ subjected to CO_2_RR for 1, 5, and 10 min (denoted as Cu_4_In_1_(1), Cu_4_In_1_(5), and Cu_4_In_1_(10)) was characterized. XRD analysis (Figure S15a, Supporting Information) indicates that as the reaction time extends, the diffraction peaks of Cu_2_O gradually weaken, and the peaks near 42.4° and 61.6° are almost completely vanishing, unfolding a reduction in Cu_2_O content within the sample. This reduction in Cu_2_O content indicates that Cu^+^ is progressively consumed during the reaction, which is consistent with the results from Cu LMM Auger spectroscopy (Figure S15b, Supporting Information). As the reaction time extends, the Cu^+^ content decreases. Similarly, In(OH)_3_ follows a similar trend, with diffraction peaks related to In(OH)_3_ completely disappearing after 10 min of reaction. Notably, as the reaction proceeds, new characteristic peaks at 34.5° and 43.1° associated with the CuIn phase are observed, indicating the in situ formation of CuIn phase during CO_2_RR.^[^ ^27^, ^37^ ^]^ To further confirm this finding, HRTEM characterization was performed on the Cu_4_In_1_(1), Cu_4_In_1_(5), and Cu_4_In_1_(10) samples (Figure S16, Supporting Information). The CuIn (200) plane is determined in the Cu_4_In_1_(5), and Cu_4_In_1_(10) samples, indicating the formation of the CuIn phase. The presence of the CuIn phase plays a pivotal role in the electroreduction of CO_2_ to CO. This is the reason why the catalytic effect of pristine **Cu** and **In** on CO is inferior, as they are incapable of forming an effective CuIn phase.

### 2.3 Electrochemical Test for Cu_4_In_1_/BP‐53

**Figure 3:**
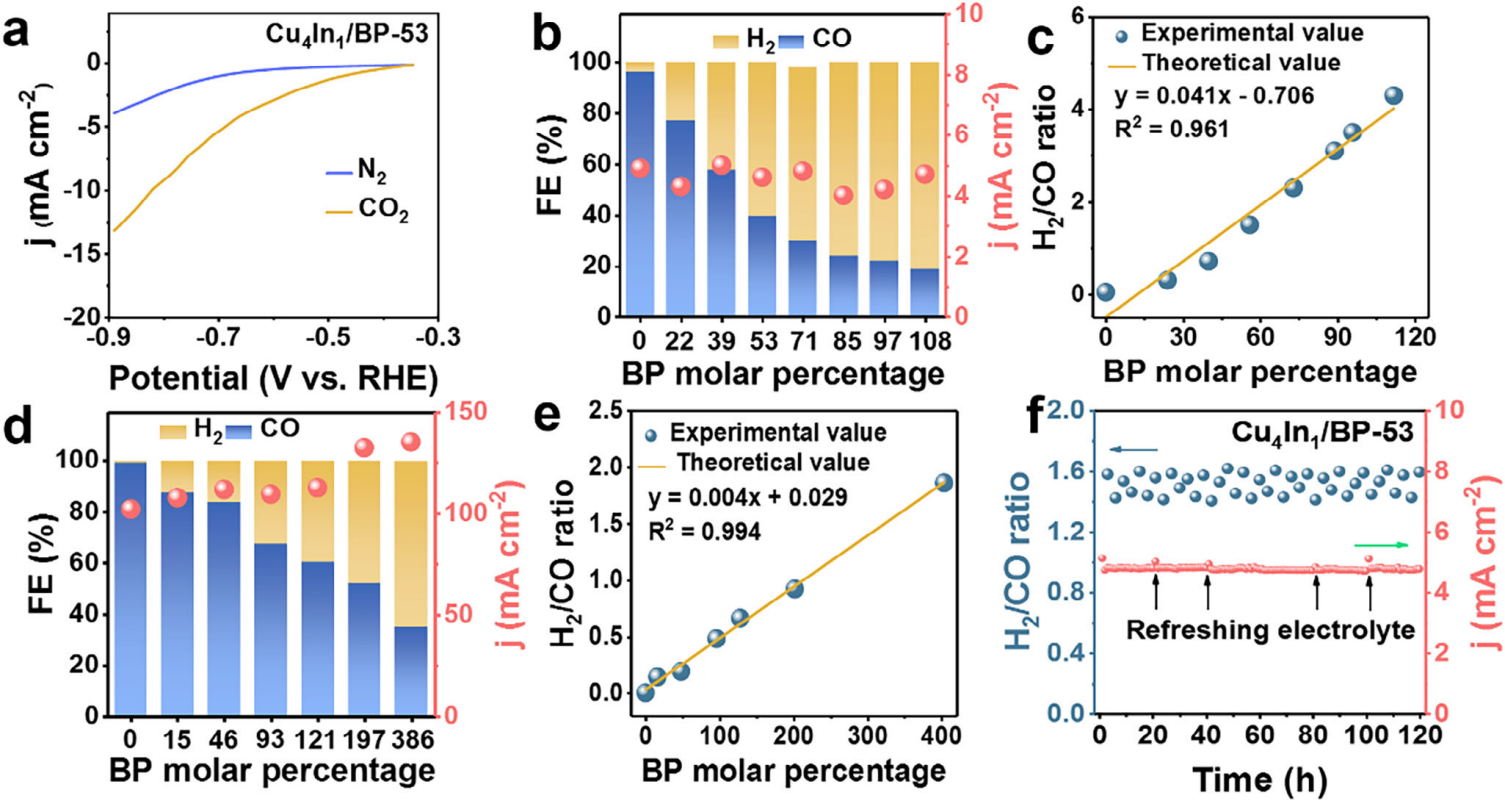
(a) LSV curves of Cu_4_In_1_/BP‐53 in N_2_‐saturated and CO_2_‐saturated electrolytes. (b) Faraday efficiencies of CO and H_2_ for Cu_4_In_1_/BP with varying BP contents at −0.6 V versus RHE. (c) Fitted linear relationship between BP content in Cu_4_In_1_/BP and H_2_/CO molar ratio at −0.6 V versus RHE. (d) Faraday efficiencies of CO and H_2_ for Cu_4_In_1_/BP with varying BP contents in a flow cell. (e) Corresponding fitted curve of BP content in Cu_4_In_1_/BP versus H_2_/CO molar ratio (at −0.6 V versus RHE). (f) H_2_/CO molar ratio is ≈1.5:1 for Cu_4_In_1_/BP‐53 during an 120 h long‐term stability test at −0.6 V versus RHE.

In the above section, the H_2_/CO molar ratio catalyzed by Cu_4_In_1_ ranges from 0.03 to 0.45 across all applied potentials, which does not meet the requirements for downstream industrial production. As reported in previous studies, BP plays a decisive role in generating H_2_.^[^ ^38^ ^]^ Therefore, BP is introduced to obtain the syngas with a specific H_2_/CO molar ratio. LSV curves were measured in N_2_/CO_2_‐saturated 0.5 M KHCO_3_ electrolyte over BP, In/BP‐53, and Cu/BP‐53 to assess their activity for CO_2_RR and HER (Figure S17, Supporting Information). As shown in Figure S17a (Supporting Information), the BP sample is unable to reduce CO_2_ as deduced from almost the same current density in both electrolytes. In contrast, In/BP‐53 catalyst has a low current response (Figure S17b, Supporting Information), while Cu/BP‐53 displays a strong current response in N_2_/CO_2_‐saturated electrolyte (Figure S17c, Supporting Information). The current density of Cu/BP‐53 in N_2_‐saturated electrolyte is higher than that of **Cu** catalyst (Figure S7b, Supporting Information) indicating that BP enhances HER. Given that Cu_4_In_1_ is an excellent electrocatalyst for CO_2_RR to generate CO and BP can generate H_2_, it is reasonable to infer that combining BP with Cu_4_In_1_, the heterogeneous catalyst is possible to generate syngas with wide H_2_/CO molar ratio. To validate the above conjecture, the FE_CO_ and $FE_{H_{2}}$ over Cu_4_In_1_/BP‐53 were determined. Prior to this, the LSV curve of Cu_4_In_1_/BP‐53 was determined in N_2_/CO_2_‐saturated electrolyte to verify CO_2_RR. Obviously, the higher current density in CO_2_‐saturated solution indicates that CO_2_ is reduced over Cu_4_In_1_/BP‐53 (**Figure** **3**a). At the potential window of −0.5 to −0.9 V versus RHE, the sum of FE_CO_ and $FE_{H_{2\;}}$ remains at 100% and the H_2_/CO molar ratio is ≈1.5 (Figure S18, Supporting Information), which is a suitable ratio to produce aldehyde.^[^ ^14^ ^]^ The addition of BP promotes the production of H_2_ and leads to the satisfied H_2_/CO molar ratio in syngas, which is mainly attributed to the existence of Cu_3_P as shown in Figure 2e. The P 2p high‐resolution XPS spectra for In/BP‐53 and Cu/BP‐53 samples were measured before and after the CO_2_RR reaction to confirm the above deduction (Figure S19, Supporting Information). For the In/BP‐53 sample, In 3d and P 2p spectra only confirm the presence of In^3+^ and P‐O species, with no changes observed before and after the reaction. Since In^3+^ and P‐O are not active species, the In/BP‐53 sample displays no activity in generating H_2_ or CO. In contrast, the Cu/BP‐53 sample leads to an increased P‐Cu bonds, a result attributed to the formation of copper phosphide. To further clarify the role of Cu_3_P in catalysis, Cu_3_P sample was synthesized via conventional pyrolysis.^[^ ^39^ ^]^ As evidenced by the XRD patterns (Figure S20, Supporting Information) and XPS spectra (Figure S21, Supporting Information), the synthesized Cu_3_P exhibits identical structural and compositional characteristics to the Cu_3_P phase presented in the Cu_4_In_1_/BP‐53 composite. These comparative analyses unambiguously confirm the in situ formation of Cu_3_P within the Cu_4_In_1_/BP‐53 system. The CO_2_RR performance of Cu_3_P was subsequently evaluated, as presented in Figure S22 (Supporting Information). The hydrogen is the major products with the FE > 80%. To further identify the contribution of the in situ‐generated Cu_3_P during electroreduction, a physically mixed Cu_3_P/Cu_4_In_1_ composite was fabricated followed by evaluation of its CO_2_ electroreduction performance (Figure S23, Supporting Information). The $FE_{H_{2\;}}$ is 58.7%, indicating that Cu_3_P mainly promotes the evolution of hydrogen, which aligns well with our conjecture and conclusion reported in the literature.^[^ ^40^ ^]^ Moreover, the HER activity improvement was accompanied by a significant suppression of CO production. Further analysis demonstrated that the total Faradaic efficiency ($FE_{H_{2}}$ + FE_CO_) consistently remained below 100%, suggesting the formation of additional byproducts. This observation unequivocally demonstrates the inability of this hybrid system to achieve controlled production of pure syngas. In the Cu_4_In_1_/BP catalytic system, the in situ formed Cu_3_P and CuIn phases rather than the simple mixture serve as the determining factor for precise regulation of the H_2_/CO molar ratio in syngas production.

### 2.4 Regulation of H_2_/CO Molar Ratio in Syngas

Although the H_2_/CO ratio over Cu_4_In_1_/BP‐53 meets our requirements, we are still interested in obtaining syngas with different ratios to accommodate a variety of applications. The FE_CO_ and $FE_{H_{2}}$ over Cu_4_In_1_/BP was tested with various BP contents at −0.6 V versus RHE. The compositional analysis of gaseous products, determined by GC, is presented in Figure S24 (Supporting Information), revealing the distribution and relative concentrations of components in the effluent stream. As shown in Figure 3b, the FE_CO_ tends to decrease but the $FE_{H_{2}}$ tends to improve with the increased BP content since BP is the main factor to prompt the generation of H_2_. The sum of FE_CO_ and $FE_{H_{2}}\;$ remains at 100% implying almost no other products. Surprisingly, it is found that there is an approximately linear relationship between the molar ratio of BP in Cu_4_In_1_/BP and the H_2_/CO molar ratio in syngas at −0.6 V versus RHE (Figure 3c). Utilizing the linear curve fit, we determined that the molar percentage of BP in Cu_4_In_1_ of 0.29, 0.42, and 0.66 correspond to H_2_/CO molar ratio of 1:2, 1:1, and 2:1, respectively, which are suitable ratio to produce methanol, ethanol, and diesel.^[^ ^6^ ^]^ To substantiate these findings, Cu_4_In_1_/BP‐29, Cu_4_In_1_/BP‐42, and Cu_4_In_1_/BP‐66 samples were synthesized, as depicted in Figure S25a–c (Supporting Information). The corresponding H_2_/CO molar ratios for these samples at −0.6 V versus RHE are 1:2.0, 1.1:1, and 1.7:1, respectively, which are basically consistent with the deduced results from the fitted linear curve. The relationship between BP content and molar ratio of H_2_/CO is also determined at −0.5 V versus RHE and −0.9 V versus RHE for Cu_4_In_1_/BP, respectively, as shown in Figure S26 (Supporting Information). The linear correlation is also observed, demonstrating that the specific H_2_/CO molar ratio in syngas can be easily regulated by varying the molar ratio of BP in Cu_4_In_1_/BP. In addition, similar tests were also conducted in a flow cell (Figure 3d,e). In this case, the maximum current density reaches 135.2 mA cm^−2^, which is ≈30 times higher than that measured in the H‐type cell. The linear relationship between BP content and H_2_/CO molar ratio demonstrates that the general applicability of the method for precisely regulating the H_2_/CO ratio in syngas by adjusting the BP content in the Cu_4_In_1_/BP catalyst. The Cu_3_P and CuIn phase are responsible for the generation of H_2_ and CO, respectively, as evidenced in above section. The formation of Cu_3_P in Cu_4_In_1_/BP leads to the reduction in Cu^+^ content, resulting in the decreased content of CuIn phase. Consequently, the H_2_/CO molar ratio becomes adjustable since the formation of phosphides accelerates the HER and suppresses the generation of CO.

As an example, Cu_4_In_1_/BP‐53 was selected to investigate the stability of the electrocatalysts at −0.6 V versus RHE (Figure 3f), and good durability is observed after 120 h measurement with slight activity decay and small fluctuation in the H_2_/CO molar ratio. Moreover, the H_2_/CO molar ratio maintained between ≈1.4 and 1.6 during the whole process, and no liquid products were detected (Figure S27, Supporting Information), which is consistent with the result deduced from the fitted linear correlation. The Cu_4_In_1_/BP‐53 sample was also probed by SEM, TEM, HRTEM, XRD, and XPS after electrochemical reactions. SEM and TEM (Figure S28a,b, Supporting Information) show that the morphology after CO_2_RR reaction is basically unchanged. HRTEM (Figure S28c, Supporting Information) indicates the generation of the CuIn (200) phase. By comparing the XRD and XPS results (Figures S29 and S30, Supporting Information), the related peaks are almost not varied indicating the good stability of the Cu_4_In_1_/BP‐53 sample.

### 2.5 Screening of Other Suitable Electrocatalysts

In the above section, a robust electrocatalyst, **CuIn/BP**, for CO_2_RR to generate syngas with the tunable H_2_/CO ratio was successfully fabricated by a simple and feasible pathway. To assess the universality of this method and enrich the member of electrocatalyst family, nine additional Cu‐based bimetallic electrocatalysts were prepared by the same method with a Cu: M molar ratio of 4:1 (M = Sn, Zn, Fe, Co, Ni, Pd, Ag, Sb, and Mg). The performance of these Cu_4_M_1_ catalysts in CO_2_RR was evaluated in CO_2_‐saturated 0.5 M KHCO_3_ solution at −0.6 V versus RHE. As shown in Figure S31a (Supporting Information), only Cu_4_Sn_1_ and Cu_4_Ag_1_ display the acceptable CO selectivity, while FE_CO_ is less than 80% over other Cu_4_M_1_ electrocatalysts unfolding the inferior CO selectivity. Then, Cu_4_Sn_1_/BP and Cu_4_Ag_1_/BP were prepared with the same method to further explore their performance in syngas generation. As shown in Figure S31b,c (Supporting Information), more H_2_ is produced with increasing BP content along with tuned H_2_/CO molar ratio, which is similar to that for Cu_4_In_1_/BP. The H_2_/CO ratio in syngas could be regulated by varying the BP content in Cu_4_M_1_/BP provided that the Cu_4_M_1_ catalyst has an acceptable CO selectivity in CO_2_RR. Cu_4_In_1_/BP exhibits the widest tunable range of H_2_/CO ratios in syngas.

To testify the uniqueness of BP in tuning the H_2_/CO ratio in syngas, Cu_4_In_1_ was combined with graphitic carbon nitride (C_3_N_4_), carbon nanotube (CNT), and molybdenum disulfide (MoS_2_), respectively,^[^ ^41^, ^42^, ^43^ ^]^ to form Cu_4_In_1_/C_3_N_4_, Cu_4_In_1_/CNT, and Cu_4_In_1_/MoS_2_. As shown in Figure S32 (Supporting Information), almost no variation is observed with the enhanced content of doped materials, implying that they are not able to generate H_2_ and tune the H_2_/CO molar ratio, which is mainly attributed to the absence of Cu_3_P in these composite catalysts. Consequently, BP exhibits the uniqueness to generate H_2_ and regulate the H_2_/CO molar ratio in syngas.

### 2.6 In Situ ATR‐FTIR Measurements

**Figure 4:**
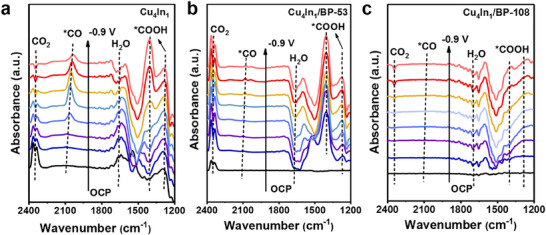
In situ ATR‐FTIR spectra of (a) Cu_4_In_1_, (b) Cu_4_In_1_/BP‐53, and (c) Cu_4_In_1_/BP‐108 during CO_2_RR in CO_2_‐saturated 0.5 M KHCO_3_ electrolyte.

To clarify the intermediates in CO_2_RR, in situ ATR‐FTIR was performed in CO_2_‐saturated 0.5 M KHCO_3_ electrolyte. The spectra were collected for Cu_4_In_1_, Cu_4_In_1_/BP‐53, and Cu_4_In_1_/BP‐108 from −0.3 to −0.9 V versus RHE with 50 mV intervals. As shown in **Figure** **4**a, the peak at 2348 cm^−1^ is associated with CO_2_. The peaks within the range of 2000–2100 cm^−1^ are assigned to the stretching vibrations of *CO intermediate adsorbed on the Cu_4_In_1_ surface. Besides, the observed peaks at 1398 and 1280 cm^−1^ are attributed to the bending and stretching vibrations of *COOH, which is generally regarded as a crucial intermediate in the formation process of *CO.^[^ ^44^ ^]^ During CO_2_RR, the peak intensity of the *CO intermediate gradually increases with the rise of applied potential, and reaches the maximum at −0.6 V versus RHE, coinciding with the maximum FE_CO_. The elevation of peak intensity reveals that the *CO and *COOH intermediates are produced during the CO_2_RR process. In brief, CO_2_ undergoes adsorption onto the Cu_4_In_1_ catalyst, and then converts into the important intermediate species, *COOH and *CO, followed by the desorption of CO from the catalyst surface. For the Cu_4_In_1_/BP‐53 catalyst, the peak intensity of *CO intermediate decreases significantly with the increase of applied potential, as shown in Figure 4b, implying that the CO production is inhibited. Furthermore, with a higher content of BP in Cu_4_In_1_/BP‐108, the peaks for both *CO and *COOH decrease (Figure 4c), suggesting a reduction in CO production.

All spectra exhibited a persistent peak at ≈1650 cm^−1^, corresponding to O‐H stretching of adsorbed water (Figure S33a–c, Supporting Information). The H_2_O molecules underwent initial activation followed by continuous consumption to supply protons for subsequent proton‐coupled electron transfer processes.^[^ ^45^ ^]^ The potential‐dependent behavior of this feature revealed distinct interfacial water dynamics. On the Cu_4_In_1_ surface, the water signal remained basically unchanged with increasingly negative potentials. While on the Cu_4_In_1_/BP‐53 surface, H_2_O progressively accumulated as evidenced by the gradual enhancement of peak intensity at more negative potentials. This observation suggests that the H_2_O consumption rate surpassed its accumulation rate on the Cu_4_In_1_/BP surface, thereby favoring *H coverage. Consequently, HER showed higher propensity on Cu_4_In_1_/BP‐53 surfaces compared to those of Cu_4_In_1_ surfaces under identical conditions. Furthermore, this effect was even more pronounced in Cu_4_In_1_/BP‐108, where the water signal showed stronger potential dependence. Therefore, the HER activity increased significantly on BP‐containing catalysts, with the H_2_ generation rate being proportional to BP content. These observations demonstrate that the H_2_/CO molar ratio in syngas can be regulated by adjusting the content of BP in the **CuIn/BP** sample.

### 2.7 DFT Calculations

To have a deeper understanding of the catalytic principle of **CuIn/BP** in CO_2_RR, DFT calculations were carried out. Two following models were built to simulate the CO_2_ conversion over Cu_4_In_1_, and Cu_4_In_1_/BP samples, respectively. Given that the In sample demonstrates negligible activity in CO_2_RR, it was excluded from our analysis.

While prior studies have emphasized the structural evolution of supported clusters and their strong substrate interactions,^[^ ^46^, ^47^ ^]^ our characterization data conclusively demonstrate that BP solely serves as a phosphorus source for in situ Cu_3_P formation without existing as free monomers in the final catalyst. This evidence justifies our exclusion of BP‐substrate interactions from the computational models. As shown in Figure S34 (Supporting Information), models representing the Cu_2_O (111) plane, CuIn (200) plane, and Cu_3_P are constructed to reflect the activity of these samples, with the active sites of them having been confirmed in previous experimental studies. To assess the stability of these samples, we calculated their formation energy (The definition is presented in supporting information) and the detailed values are provided in Table S3 (Supporting Information). A more negative formation energy indicates a higher energy release, thus enhancing the sample's stability. Among all the samples, Cu_3_P exhibits the most negative formation energy of −1.44 eV, suggesting that it is the most stable and easily formed. In contrast, CuIn (200) has the least negative formation energy of −0.71 eV, making it the most unstable and difficult to form. These computational results are consistent with the aforementioned experimental data. Notably, experimental characterization confirmed the presence of Cu_2_O phases significantly influencing catalytic outcomes. Accordingly, two additional heterojunction models were built: 1) Cu_2_O‐CuIn‐C with intermediates adsorbed at the heterojunction center, and 2) Cu_2_O‐CuIn‐S featuring simultaneous adsorption on both Cu_2_O and CuIn surfaces (Figure S35, Supporting Information). The formation energies of Cu_2_O‐CuIn‐C and Cu_2_O‐CuIn‐S are also listed in Table S3 (Supporting Information).

**Figure 5:**
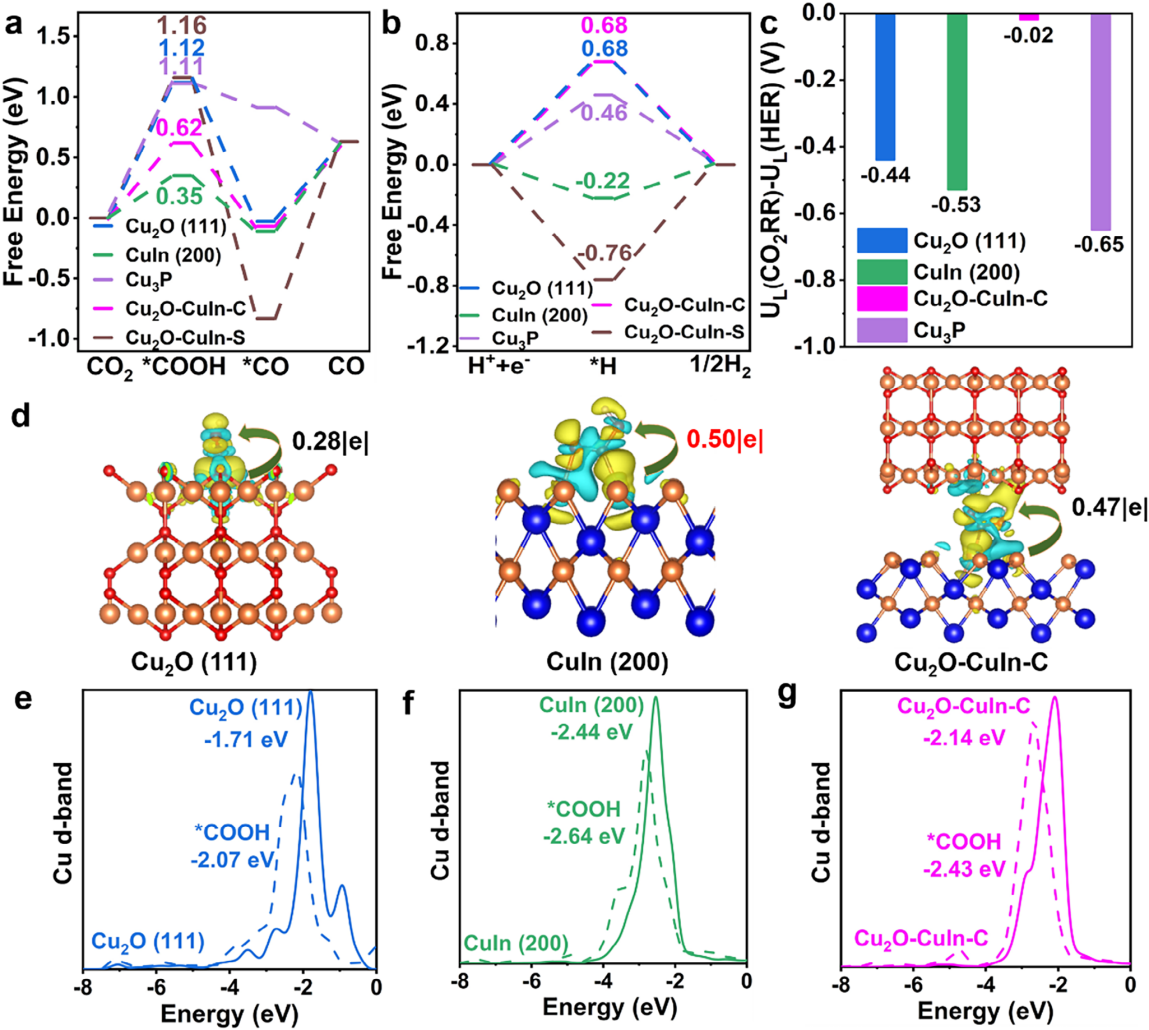
Comparative mechanistic analysis of catalytic surfaces: (a) Free energy diagrams for CO_2_RR pathways on Cu_2_O (111), CuIn (200), Cu_3_P, Cu_2_O‐CuIn‐C, and Cu_2_O‐CuIn‐S surfaces. (b) Corresponding free energy profiles for HER. (c) The overpotential difference between CO_2_RR and HER for Cu_2_O (111), CuIn (200), Cu_3_P and Cu_2_O‐CuIn‐C models. (d) Charge density difference maps showing *COOH adsorption configurations on each surface. (e–g) Electronic structure modifications upon *COOH binding: d‐band structure evolution and center shifts (numerical values indicated) for (e) Cu_2_O (111), (f) CuIn (200) and (g) Cu_2_O‐CuIn‐C surfaces.

Free energy calculations for CO_2_RR on all five models (**Figure** **5**a) confirmed the established two‐electron transfer pathway,^[^ ^48^, ^49^ ^]^ with *COOH formation as the rate‐determining step (RDS). The CuIn (200) surface exhibited the lowest RDS energy variation (0.35 eV), suggesting superior CO generation capability compared to other models. Given our focus on syngas production, we further analyzed HER activities (Figure 5b). The limiting potential differences between CO_2_RR and HER (Figure 5c) revealed Cu_3_P has the most negative potential, indicating the strongest hydrogen evolution tendency. This potential difference serves as a quantitative descriptor – more negative values favor H_2_ production while less negative values promote CO formation. By precisely controlling BP content in CuIn/BP, we can tune Cu_3_P formation and consequently regulate the H_2_/CO molar ratio, in excellent agreement with our experimental results. Overall, **CuIn/BP** provides an improved balance between CO_2_RR and HER activities to tune the H_2_/CO molar ratio, which is consistent with our experimental findings.

The detailed structural information and charge distribution of *COOH on the above samples were further analyzed. As illustrated in Figure S36 (Supporting Information), Cu_2_O (111) and Cu_2_O‐CuIn‐C exhibit single‐adsorption configurations, while CuIn (200) adopts a dual‐adsorption configuration. The lower *COOH binding energy on CuIn (200) is attributed to its dual‐adsorption nature. The larger Cu‐O‐C bond angle (100°) observed on the CuIn (200) surface alleviates repulsion between the intermediates, resulting in a stable Cu‐O bond with a bond length of 2.04 Å. In contrast, the smaller Cu‐O‐C angles (84° and 75°) on the surfaces of Cu_2_O (111) and Cu_2_O‐CuIn‐C lead to longer and less stable Cu‐O bonds (3.40 and 4.06 Å), hindering the formation of a stable doubly adsorbed *COOH. Consequently, the adsorption of *COOH on CuIn (200) is more stable, which is in line with its lower free energy in CO_2_RR. Figure 5d presents the charge density differences for *COOH adsorbed on the Cu_2_O (111) plane, CuIn (200) plane, and Cu_2_O‐CuIn‐C. The CuIn (200) plane donates more electrons to *COOH than the other models. The strong electronic interaction enhances CO_2_ molecule activation and *COOH intermediates stabilization, significantly boosting CO_2_RR activity. Figure 5e–g presents a comprehensive analysis of d‐band structure modifications and center shifts for reactive Cu sites on Cu_2_O (111), CuIn (200), and Cu_2_O‐CuIn‐C surfaces. The d‐band centers are located at −1.71, −2.44, and −2.14 eV for the three samples, respectively. After *COOH adsorbs on the surface during the CO_2_RR, the d‐band center of Cu_2_O (111) shifts from −1.71 to −2.07 eV, while that of Cu_2_O‐CuIn‐C shifts from −2.14 to −2.43 eV. These shifts are larger than that of CuIn (200) (from −2.44 to −2.64 eV). The minimal electronic structure changes imply that Cu active sites are stabilized by interactions with In, explaining the enhanced CO_2_‐to‐CO performance of CuIn (200). However, the electrocatalytic performance depends not only on *COOH adsorption but also on *CO desorption. An optimal balance between *COOH adsorption and *CO desorption is critical for electrocatalyst design. As shown in Figure S37 (Supporting Information), Cu_2_O‐CuIn‐C exhibits the best performance due to its intermediate d‐band center position and moderate shift after *COOH adsorption. Besides the above items, structural reconstruction and edge‐site activation will also influence catalytic activity,^[^ ^50^, ^51^ ^]^ however, these aspects fall beyond the scope of the current investigation. A comprehensive examination of these factors will be addressed in our future studies.

## 3 Conclusion

The heterostructure catalyst **CuIn/BP**, synthesized via a straightforward one‐step co‐reduction method, demonstrates an exceptional ability to precisely tune the CO/H_2_ molar ratio during the electroreduction of CO_2_ to syngas. This catalyst achieves a current density of 135.2 mA cm^−2^ in a flow cell at −0.6 V versus RHE along with an ≈100% FE for H_2_ and CO production. An important finding is the linear relationship between the BP content in **CuIn/BP** and the resulting H_2_/CO molar ratio. This relationship enables the efficient production of syngas with a tailored H_2_/CO composition suitable for various industrial applications. The CuIn phase and Cu_3_P species are formed in situ in the **CuIn/BP** catalyst, where the CuIn phase primarily facilitates CO production, while the Cu_3_P species enhances H_2_ generation, thus allowing for the precise adjustment of the H_2_/CO ratio in syngas, as confirmed by in situ ATR‐FTIR and DFT calculations. Thus, this work not only presents a new catalyst for CO_2_ electroreduction to syngas with a desired H_2_/CO ratio, but also reveals the possible mechanism behind its excellent electrocatalytic performance.

## Conflict of Interest

The authors declare no conflict of interest.

## Supporting information



Supporting Information

## Data Availability

The data that support the findings of this study are available from the corresponding author upon reasonable request.
