# Supplementary material for: An Innovative Approach to Precisely Tailor the Composition of Syngas in CO2 Electroreduction
Source: Adv Sci (Weinh). 2025 Jun 23;12(35):e05424. doi: 10.1002/advs.202505424 (PMC12462950; doi:10.1002/advs.202505424)
Supplement: Supplementary file 1 — Supporting Information [file ADVS-12-e05424-s001.docx]

Supporting Information

**A Innovative Approach to Precisely Tailor the Composition of Syngas in CO_2_ Electroreduction**

*Shuai Lv^†^, Xinyi Sun^†^, Baolin Wang, Wenrui Wan, Li Wang^*^, Jianji Wang^*^, Jinglai Zhang^*^*

^†^ Shuai Lv and Xinyi Sun contributed equally to this work

S. Lv, X. Sun, B. Wang, L. Wang, J. Zhang

Henan Key Laboratory of Protection and Safety Energy Storage of Light Metal Materials

College of Chemistry and Molecular Sciences

Henan University

Kaifeng, Henan 475004, P. R. China

E-mail: [chemwangl@henu.edu.cn](mailto:chemwangl@henu.edu.cn); [zhangjinglai@henu.edu.cn](mailto:zhangjinglai@henu.edu.cn)

W. Wan, J. Wang

Key Laboratory of Green Chemical Media and Reactions (Ministry of Education)

Collaborative Innovation Centre of Henan Province for Green Manufacturing of Fine Chemicals

School of Chemistry and Chemical Engineering

Henan Normal University

Xinxiang, Henan 453007, P. R. China

E-mail: [jwang@htu.edu.cn](mailto:jwang@htu.edu.cn)

**Chemicals.** All chemicals were used as received without additional purification. Copper (II) nitrate trihydrate (Cu(NO_3_)_2_·3H_2_O, AR, Aladdin), indium (III) nitrate hydrate (In(NO_3_)_3_·xH_2_O, 99.99%, Aladdin), copper (II) chloride dihydrate (CuCl_2_·2H_2_O, AR, Aladdin), tin (IV) chloride (SnCl_4_, AR, Aladdin), zinc nitrate hexahydrate (Zn(NO_3_)_2_·6H_2_O, AR, 99%, Aladdin), iron (III) chloride (FeCl_3_, AR, Aladdin), cobalt(II) chloride (CoCl_2_, 97%, Aladdin), nickel (II) nitrate hexahydrate (Ni(NO_3_)_2_·6H_2_O, AR, 98%, Aladdin), copper (II) acetate monohydrate (Cu(CH_3_COO)_2_·H_2_O, 99%, Aladdin), palladium (II) acetate (Pd(CH_3_COO)_2_, AR, Aladdin), silver nitrate (AgNO_3_, AR, 99.8%, Aladdin), antimony (III) acetate (Sb(CH_3_COO)_3_, 97%, Aladdin), magnesium chloride (MgCl_2_, 99.9%, Aladdin), sodium borohydride (NaBH_4_, 98%, Innochem), potassium bicarbonate (KHCO_3_, AR, 99.5%, Aladdin), potassium hydroxide (KOH, AR, 85%, Aladdin), ethanol (≥99.5%, Fuyu Fine), bulk black phosphorus (BP, >99.998%, Hefei Keliao), Nafion solution (5 wt.% in a mixture of lower aliphatic alcohols and water, Sigma-Aldrich), isopropyl alcohol (≥99.5%, Macklin), carbon paper (HCP020P, HESEN), gas diffusion layer (GDL, YLS-30T, HESEN), ultrapure water, melamine (C_3_H_3_N_6_, 99%, Aladdin), molybdenum disulfide (MoS_2_, 98%, Aladdin), carbon nanotubes (CNT, >95%, Aladdin), hydrochloric acid (37%, Fuyu Fine), tetrabutylammonium hexafluorophosphate (TBAP, 98%, Aladdin), N,N-dimethylformamide (DMF, 99.5%, Fuyu Fine).

**Preparation of BP nanosheets.** BP nanosheets were prepared via an electrochemical exfoliation method. Typically, ~2 g of bulk BP crystal were attached to an electrode through a clamp. Ni foam was used as the counter electrode, and the electrolyte was prepared by dissolving TBAP (2 g) in DMF (40 mL). After the bulk BP electrode was immersed into the electrolyte, a -12 V potential was applied using a DC power supply. The bulk BP quickly swelled because of the insertion of TBA^+^ ions leading to the detachment of BP fragments, and indicating successful exfoliation of black phosphorus nanosheets. The exfoliation ended when the current stopped decreasing. Then, the unexfoliated BP crystal were removed, and the DMF solution containing BP nanosheets was collected and ultrasonicated for 4 h to ensure thorough dispersion. To obtain ultrathin BP nanosheets, the dispersion was centrifuged for 10 min at 2000 rpm and the precipitate was removed. The BP nanosheets remaining in DMF after slow centrifugation was kept in DMF at a concentration of 0.16 mmol mL^-1^ for further use.

**Synthesis of Cu-based bimetallic samples and CuM/BP.** A series of Cu-based bimetallic catalysts were prepared by a one-step co-reduction method using NaBH_4_ with various metal precursors. The specific precursors used for each sample are detailed in Table S1. In detail, the total molar mass of two precursors was fixed to 2 mmol. The precursor was dispersed into 20 mL of ultrapure water, then, 10 mL of 10 mM NaBH_4_ solution was slowly added drop by drop to form a black precipitate, which was stirred violently until the supernatant was colorless. The product was centrifuged in ethanol and ultrapure water (8000 rpm, 5 min). Finally, the samples were dried under vacuum at 60 °C for 12 h. The synthesis of **CuM/BP** is similar to above procedure except that a small amount of DMF solution containing BP nanosheets was added to the mixed solution with metal precursors in the initial stage. The samples are named according to the proportion of bimetallic precursors and the quantity of BP added. For example, Cu_4_In_1_/BP-53 means that a catalyst with a Cu to In precursor molar ratio of 4:1 and the molar percentage of BP to **CuIn** is 0.53 %, where 53 represents 0.53 expanded 100 times.

**Electrochemical measurements.** To construct the working electrode, the catalyst slurry containing 480 μL isopropanol, and 20 μL Nafion solution and 5 mg of obtained catalyst was mixed and sonicated. Then, the catalyst slurry was slowly dripped onto the surface of carbon paper or GDL until the loading capacity was 2 mg cm^-2^. All electrochemical measurements were performed on CHI650E electrochemical workstation using a three-electrode system. The potential range of electrolysis was -0.5 V to -0.9 V vs. RHE with a step interval of 0.1 V. For H type cell, 0.5 M KHCO_3_ aqueous solution as cathode and anode electrolyte, Pt mesh and Hg/HgO electrode as the counter and reference electrode, respectively. Throughout the CO_2_RR process, CO_2_ was continuously bubbled into the cathode compartment to ensure sufficient supply. The pH value of CO_2_-saturated 0.5 M KHCO_3_ electrolyte was measured to be 7.2. The potentials were converted to the RHE using the Nernst equation: E_RHE_ = E_SCE_ + 0.244 + 0.0591 × pH. For flow cell, Ni foam and Ag/AgCl electrode as the counter and reference electrodes, respectively. 1 M KOH was used as the electrolyte, and was circulated through the cathodic and anodic chambers using peristaltic pumps. The flow rate of CO_2_ gas through the gas chamber was kept constant at 20 mL min^–1^ using a digital gas flow controller. The pH value of CO_2_-saturated 1 M KOH electrolyte was measured to be 14. The potentials were converted to the RHE using the Nernst equation: E_RHE_ = E_Ag/AgCl_ + 0.210 + 0.0591 × pH. The composition of gas-phase products was analyzed by using an online gas chromatograph (GC, PANNA A91 Plus) for 15 min. The detectors for H_2_ and CO are thermal conductivity detector and flame ionization detector, respectively. The FEs of gas-phase products (CO and H_2_) were calculated by the following expression:

$$\text{FE=}\frac{\text{nzF}}{\text{Q}}\text{=}\frac{\text{V\%}\text{υ}\text{zF}}{\text{V}_{\text{m}}\text{I}}\text{×100\%}$$

where V% is the relative gas content; z is the number of electrons required for reduction of CO_2_ to CO, $\text{υ}$ is the CO_2_ flow rate, F is Faraday’s constant (96485 C mol^−1^), and V_m_ is the gas molar volume, V_m_ = 22.4 L mol^-1^ under standard conditions, I is the current density. The nuclear magnetic resonance (NMR, Bruker BioSpin GmbH 600 MHz) was used for the detection of liquid product. A 0.5 mL sample of the KHCO_3_ solution after CO_2_RR was mixed with the addition of 0.1 mL D_2_O and then transferred to the NMR sample tube. The water suppression method was used.

**Characterizations.** The morphology and structure of the catalysts were examined by scanning electron microscopy (SEM, JSM-7610F). Observation of the internal structure of the material and images of the distribution of each element were performed by transmission electron microscopy (TEM, JEM-F200). X-ray diffraction (XRD) was measured by a Bruker AXS D8 Advance diffractometer with a diffraction source of Cu Kα (λ = 1.5418Å) and a scanning range of 20° ~ 70°. X-ray photoelectron spectroscopy (XPS) was performed on a Thermo Scientific Escalab-250Xi electron spectrometer using an Al Kα X-ray source, all test results were calibrated with the C 1s peak at 284.8 eV as an internal standard. The element molar content of as-prepared catalysts was determined by inductively coupled plasma atomic emission spectroscopy (ICP-OES) on Agilent ICP-OES 725 ES.

***In situ* ATR-FTIR measurements.** The in situ attenuated total reflectance-Fourier transform infrared spectroscopy (in situ ATR-FTIR) were obtained on a BRUKER VERTEX 70v spectrometer cooled by liquid nitrogen. The measurements were performed in a custom-built H-cell with CO_2_ saturated 0.5 M KHCO_3_ aqueous solution as the electrolyte. The gold-plated silicon prism was used to load different catalysts. A Pt mesh electrode and an Ag/AgCl electrode were used as counter and reference electrodes, respectively. All spectra were collected at a resolution of 4 cm^-1^, with an average of 200 scans per spectrum. High-purity CO_2_ was continuously introduced into the electrolyte during the reaction.

**Computation section.** All calculations were performed using the spin-polarized density functional theory (DFT) implemented in the Vienna Ab-initio Simulation Package (VASP).^[1, 2]^ The Perdew-Burke- Ernzerhof (PBE) functional with generalized gradient approximation was used to describe the electron exchange correlation energy.^[3, 4]^ The plane-wave truncation energy was set to 500 eV, and the convergence criteria for the electron energy and force were set to 10^-5^ eV and 0.05 eV Å^-1^. A k-point of 12 × 12 × 1 was used in the partitioned density of states (PDOS) calculation.^[5]^ To avoid interactions between the periodic cells, a 20 Å vacuum layer was introduced in the z-direction. The thermodynamic free energy is defined as following:^[6]^

$$\text{∆}\text{G}\text{ }\text{=}\text{ }\text{∆}\text{E}\text{ }\text{+}\text{ }{\text{∆}\text{E}}_{\text{ZPE}}\text{ }\text{+}\text{ }\int\text{CpdT}\text{ }\text{-}\text{ }\text{T∆S}\text{ }\text{+}\text{ }\text{∆}\text{GpH}$$

Among them, ΔE is the change of reaction energy calculated by DFT, ΔE_ZPE_, $\int\text{CpdT}$ and TΔS are the change of zero points energy, the enthalpy changes from 0 to TK, and the entropy change at 298K. ∆GpH is the free energy correction of pH, which can be expressed as $\text{∆}\text{GpH}\text{ = 2.303}\text{ }\text{kB}\text{ }\text{T }\text{pH}$. In this paper, the effect of pH is not considered, so the pH value is set to 0. The limiting potential (U_L_) was defined as:

$$\text{U}_{\text{L}}\text{=-}\frac{\text{∆}\text{G}_{\text{max}}}{\text{e}}$$

in which ΔG_max_ is the maximum free energy change among all of the elementary steps. The formation energy of Cu (111) metal nanoparticles is calculated according to formula (1):^[7, 8]^

$\text{E}_{\text{b}}\text{=}{\text{E}_{\text{Cu}}}/\text{n}\text{-}\text{E}_{\text{Cu}}$ (1)

where E_Cu_ is the calculated lowest energies of Cu metal nanoparticles total structures. The variables n denote the number of Cu atoms. The formation energy of CuIn (200), Cu_2_O (111) and Cu_3_P metal

nanoparticles is calculated in formula (2), (3), (4):

$\text{E}_{\text{b}}\text{=}\left( \text{E}_{\text{CuIn}}\text{-}\text{n}_{\text{1}}\text{E}_{\text{Cu}}\text{-}\text{n}_{\text{2}}\text{E}_{\text{In}} \right)/\left( \text{n}_{\text{1}}\text{+}\text{n}_{\text{2}} \right)$ (2)

$\text{E}_{\text{b}}\text{=}\left( \text{E}_{\text{Cu}_{\text{2}}\text{O}}\text{-}\text{n}_{\text{1}}\text{E}_{\text{Cu}}\text{-}\text{n}_{\text{2}}\text{E}_{\text{O}} \right)/\left( \text{n}_{\text{1}}\text{+}\text{n}_{\text{2}} \right)$ (3)

$\text{E}_{\text{b}}\text{=}\left( \text{E}_{\text{Cu}_{\text{3}}\text{P}}\text{-}\text{n}_{\text{1}}\text{E}_{\text{Cu}}\text{-}\text{n}_{\text{2}}\text{E}_{\text{P}} \right)/\left( \text{n}_{\text{1}}\text{+}\text{n}_{\text{2}} \right)$ (4)

where E_Cu_, E_In_, E_O_ and E_P_, represent the energies of individual Cu atoms, In atoms, O atoms and P atoms. E_CuIn_, $\text{E}_{\text{Cu}_{\text{2}}\text{O}}$, and $\text{E}_{\text{Cu}_{\text{3}}\text{P}}$ is the calculated lowest energies of CuIn metal nanoparticles, Cu_2_O metal nanoparticles, and Cu_3_P metal nanoparticles total structures. The variables n_1_ and n_2_ denote the number of atoms of each type involved.


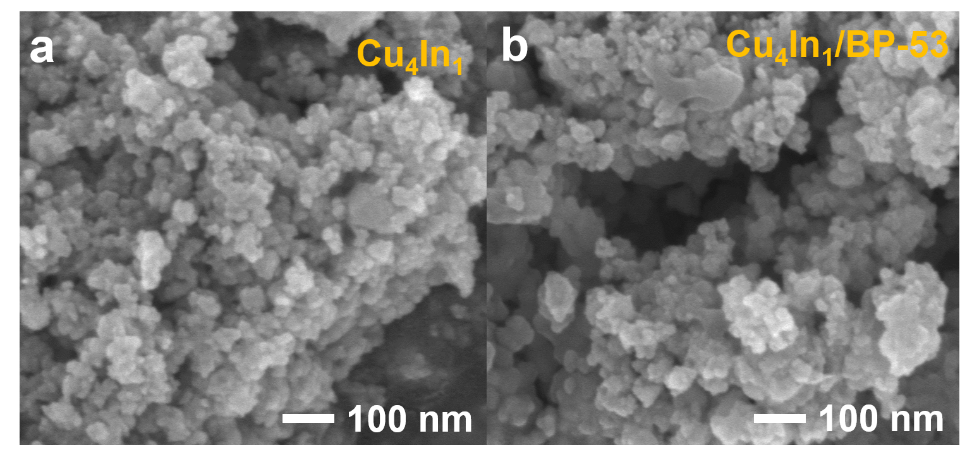


**Figure S1.** SEM images of (a) Cu_4_In_1_ (a) and (b) Cu_4_In_1_/BP-53.


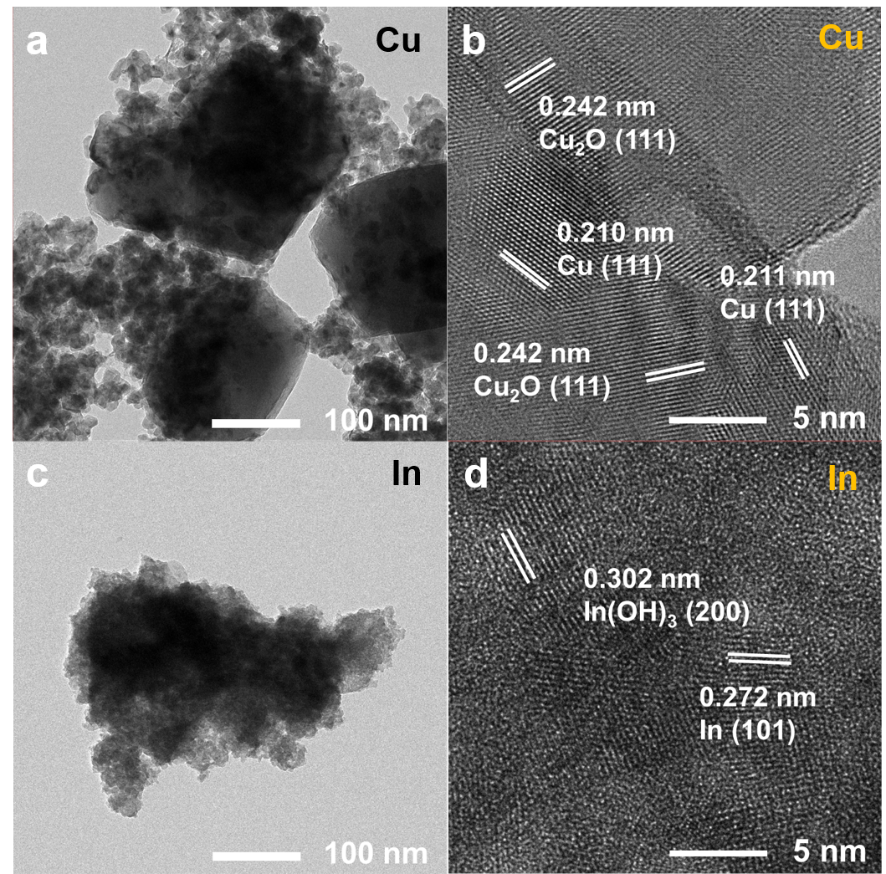


**Figure S2.** TEM images of **Cu** sample (a) and **In** sample (c). HRTEM images of of **Cu** sample (b) and **In** sample (d).


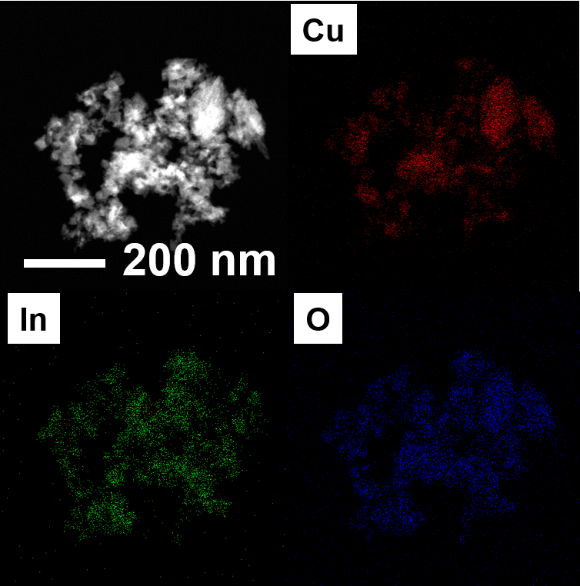


**Figure S3.** EDS mappings of Cu_4_In_1_.


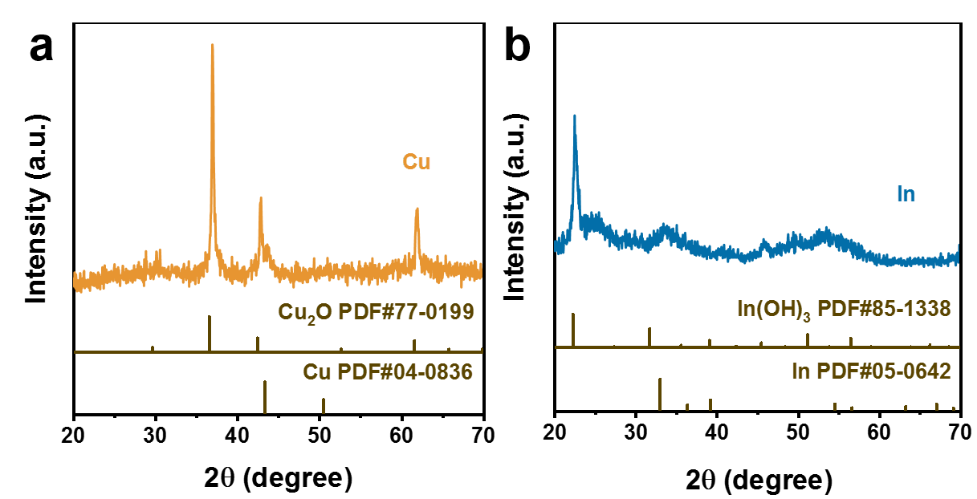


**Figure S4.** XRD patterns of (a) **Cu** sample and (b) **In** sample.


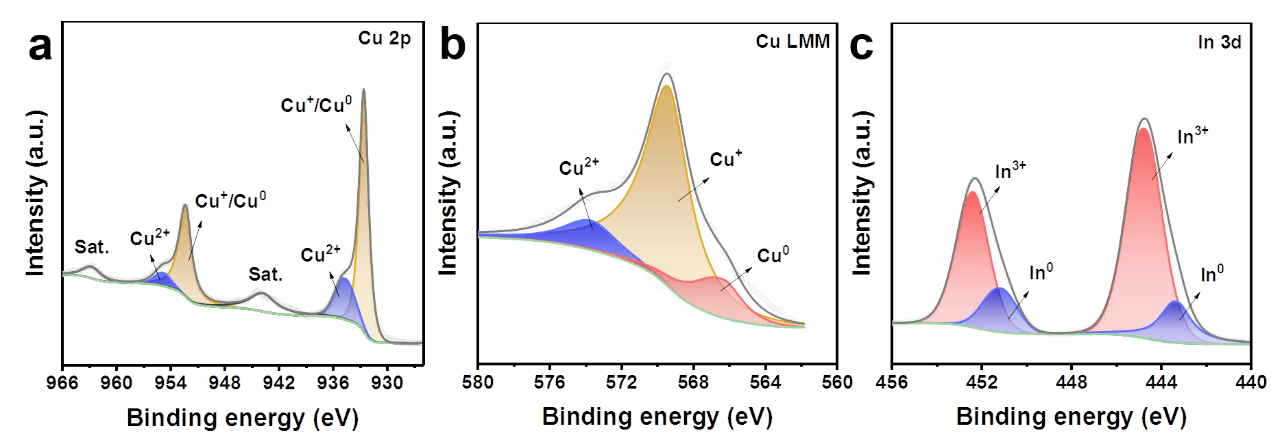


**Figure S5.** XPS spectra of **Cu** sample: (a) Cu 2p and (b) Cu LMM auger. (c) In 3d XPS spectra of **In** sample.


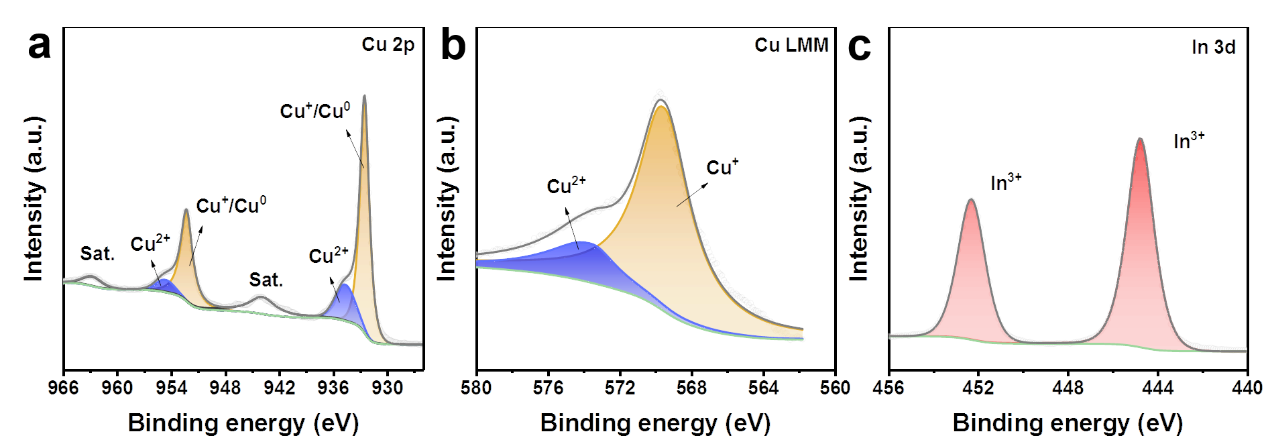


**Figure S6.** XPS spectra of Cu_4_In_1_: (a) Cu 2p, (b) Cu LMM and (c) In 3d.


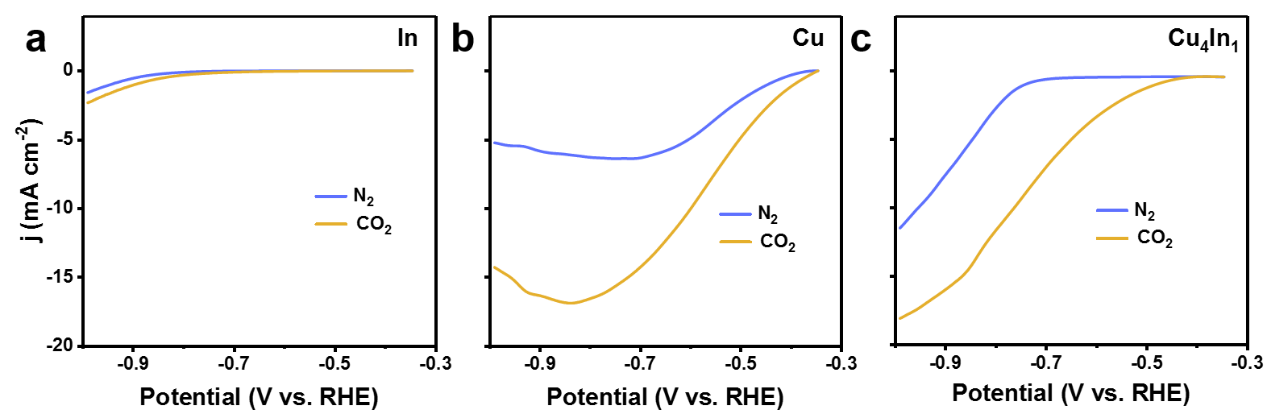


**Figure S7.** The LSV curves under N_2_/CO_2_-saturated 0.5 M KHCO_3_ electrolyte: (a) **In**, (b) **Cu** and (c) Cu_4_In_1_.


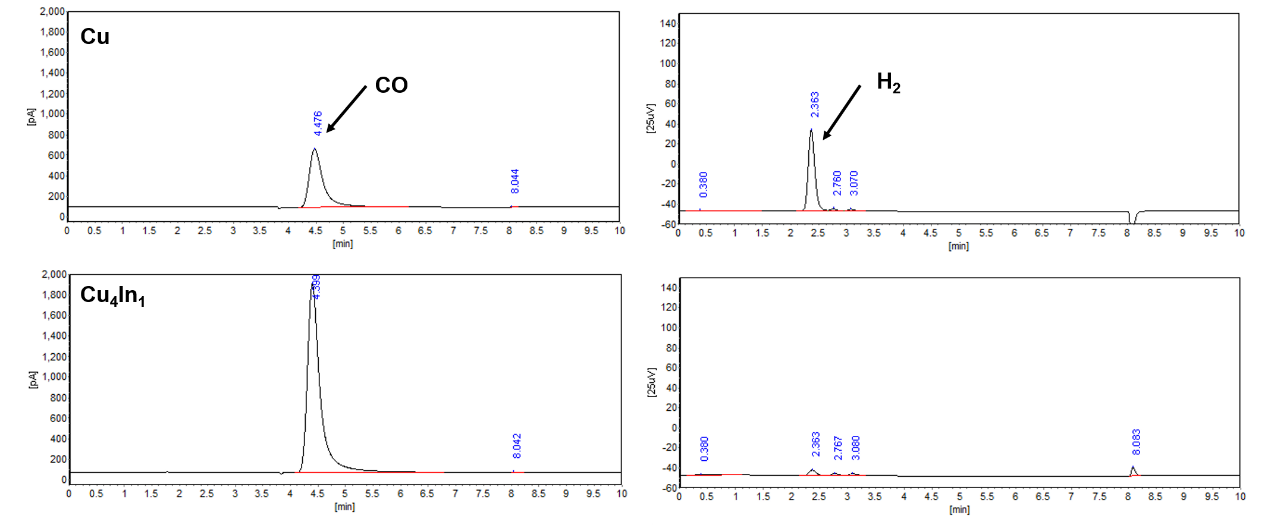


**Figure S8.** Representative gas-chromatography data of gas products of **Cu** and Cu_4_In_1_ sample.


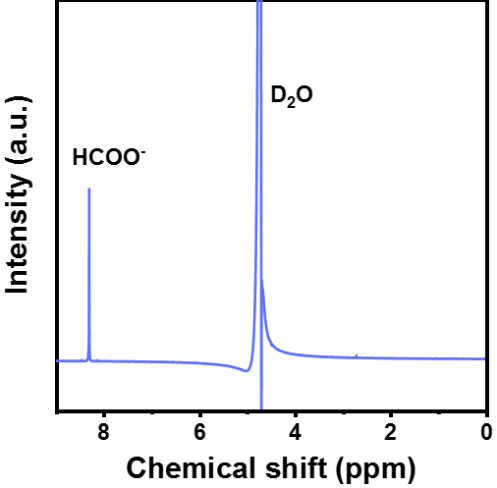


**Figure S9.** ^1^H NMR spectroscopy of liquid products.


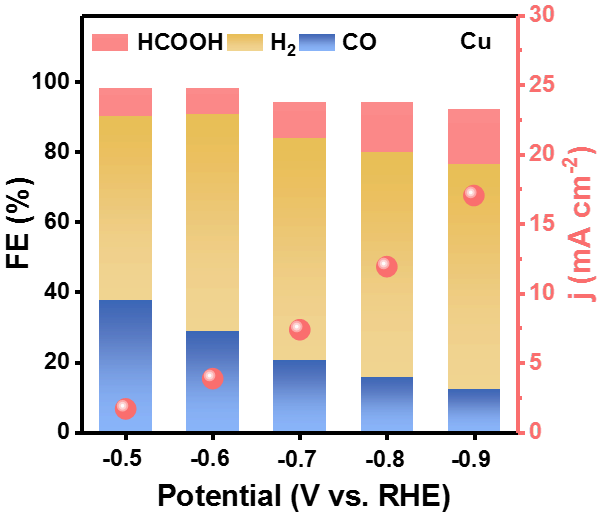


**Figure S10.** CO_2_RR performances of Cu sample under different potentials.

**
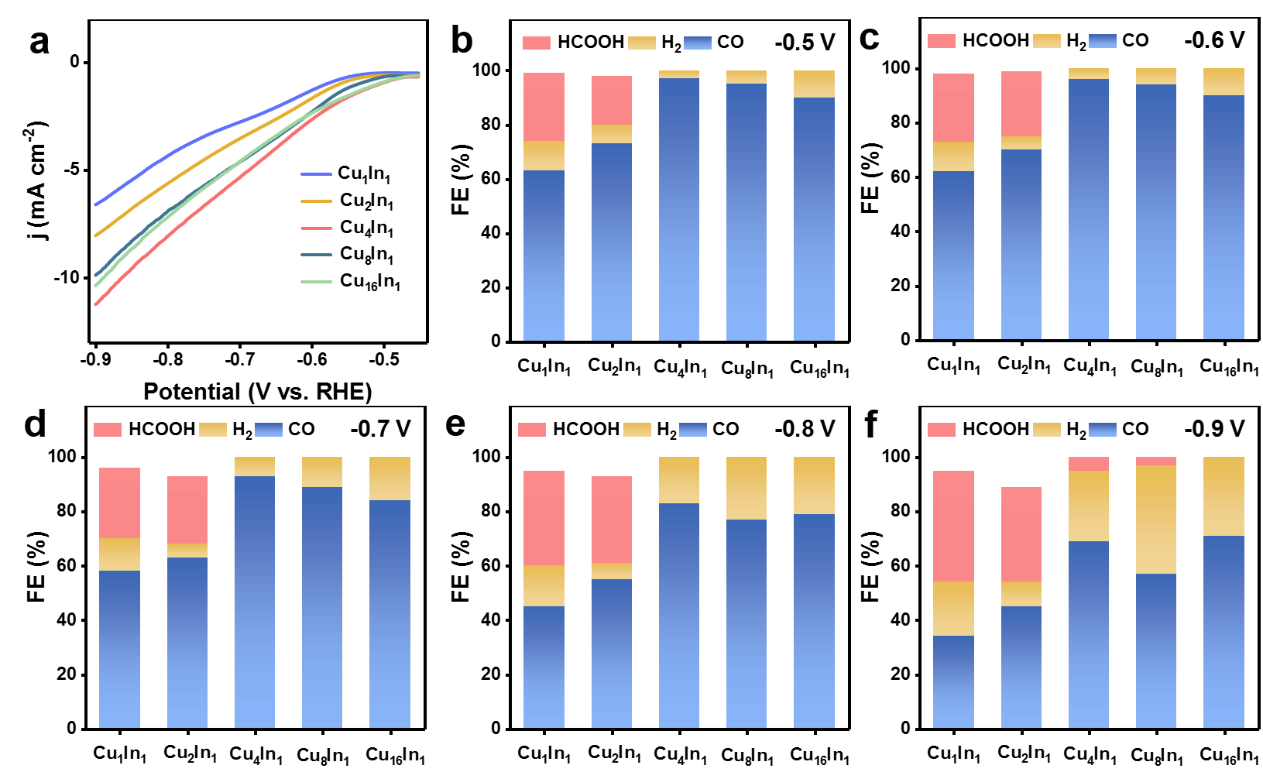
**

**Figure S11.** (a) LSV curves of different Cu_x_In_1_ samples under CO_2_-saturated 0.5 M KHCO_3_ electrolyte. CO_2_RR performance of different Cu_x_In_1_ samples: (b) at -0.5 V vs. RHE, (c) at -0.6 V vs. RHE, (d) at -0.7 V vs. RHE, (e) at -0.8 V vs. RHE, (f) at -0.9 V vs. RHE.


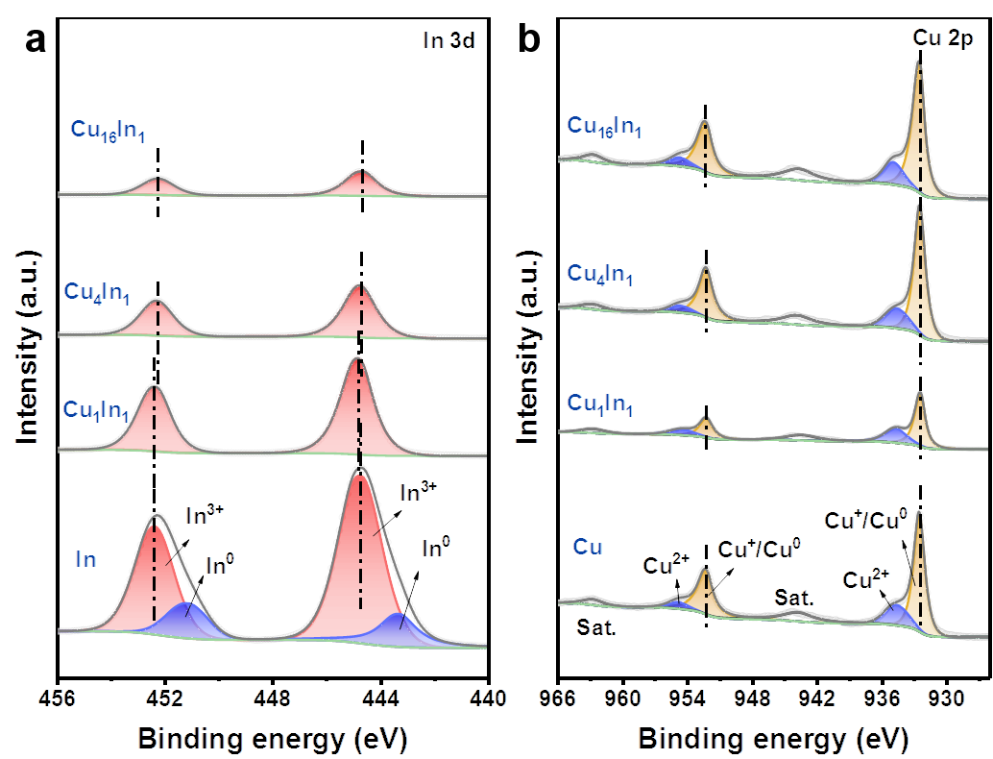


**Figure S12.** XPS spectra of **CuIn** samples with different Cu/In ratios: (a) In 3d and (b) Cu 2p.


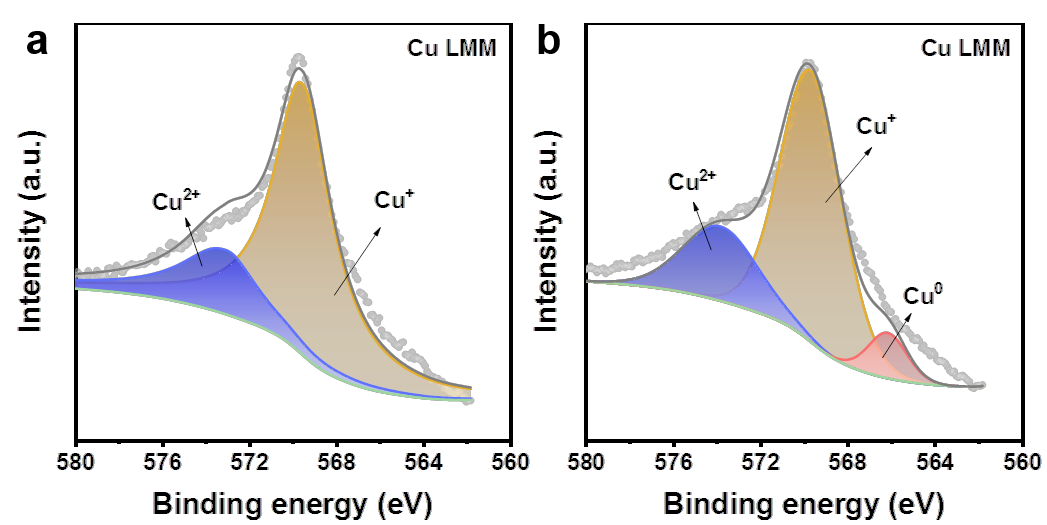


**Figure S13.** Cu LMM auger spectra of (a) Cu_1_In_1_ and (b) Cu_16_In_1_.


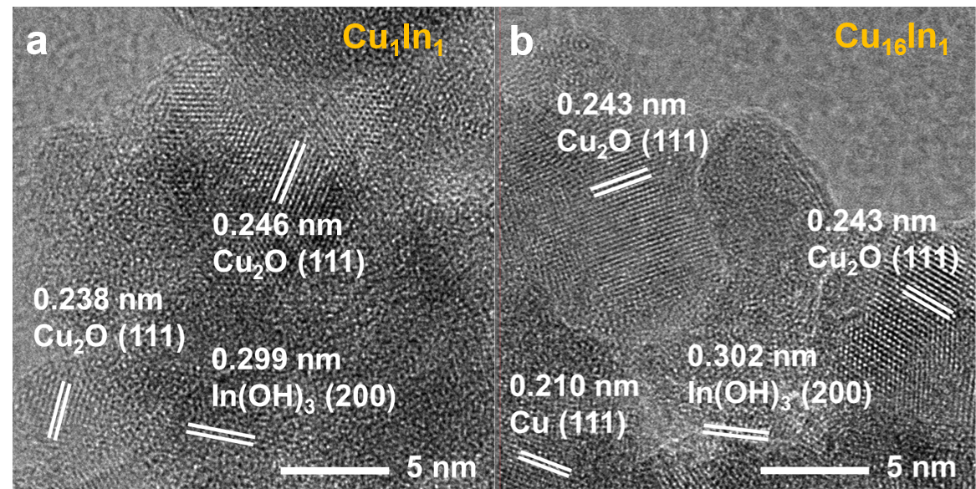


**Figure S14.** HRTEM image of (a) Cu_1_In_1_ and (b) Cu_16_In_1_.


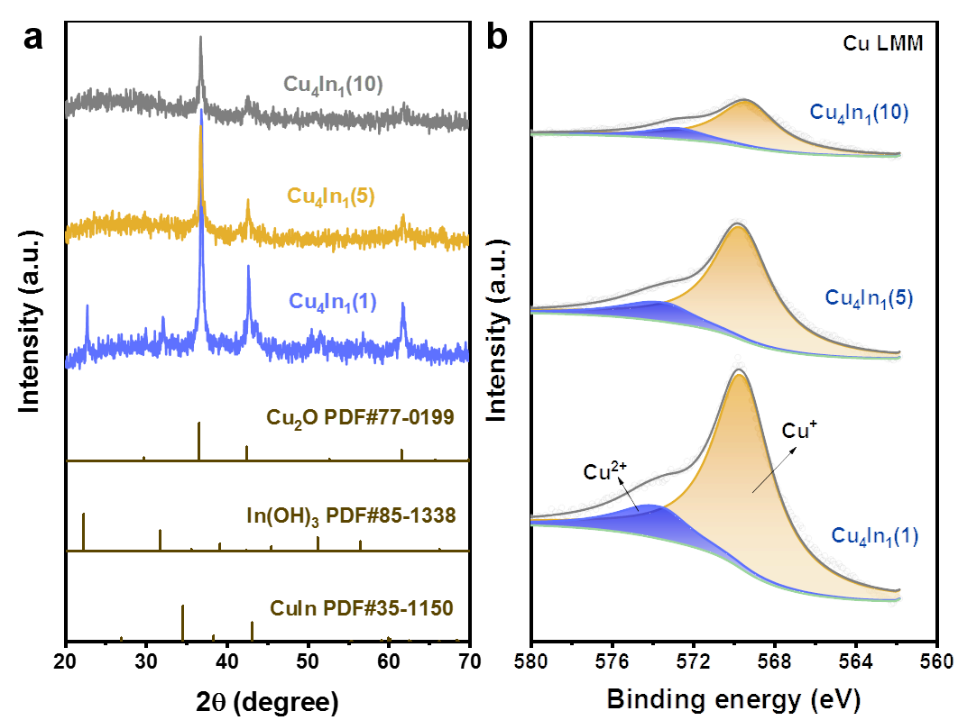


**Figure S15.** (a) XRD patterns of Cu_4_In_1_ sample with different reaction times. (b) Cu LMM auger spectra of Cu_4_In_1_ sample with different reaction time.


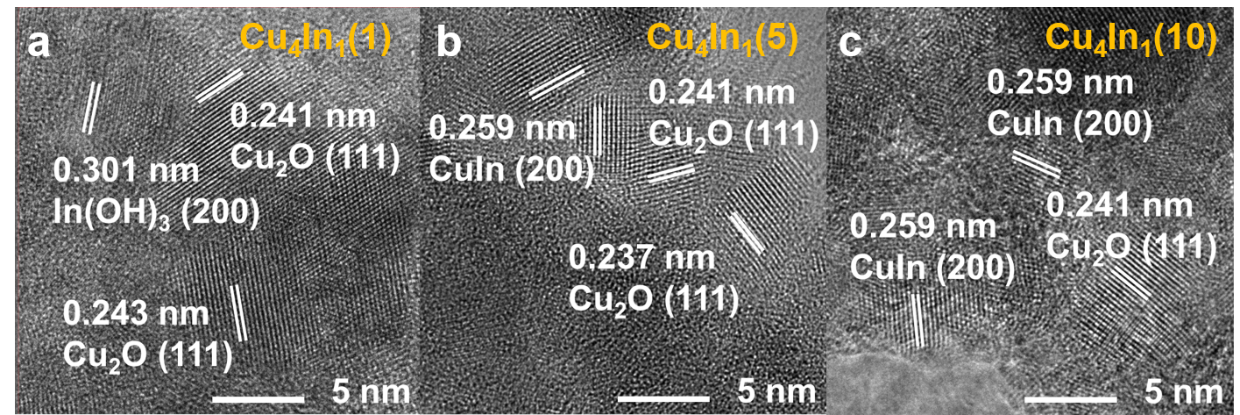


**Figure S16.** HRTEM images of Cu_4_In_1_ sample with different reaction time.


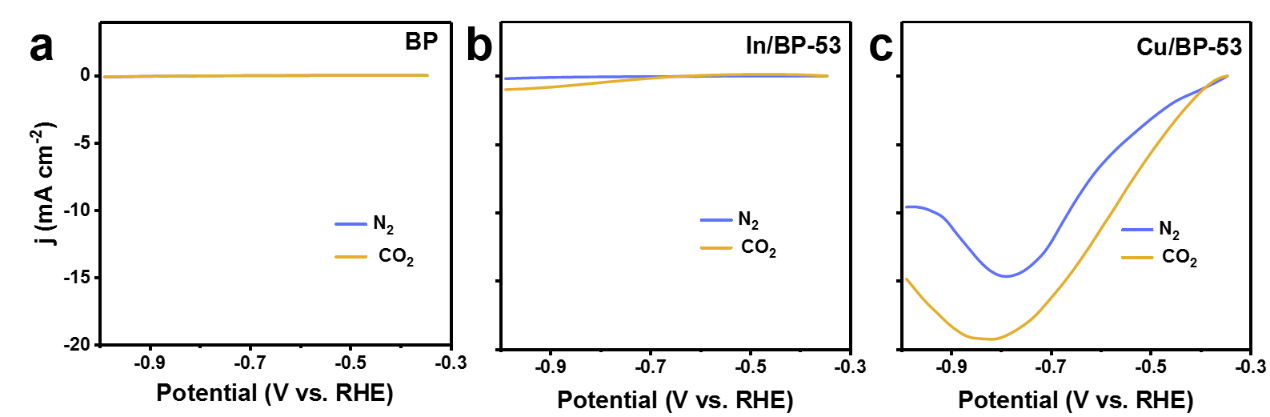


**Figure S17.** The LSV curves under N_2_/CO_2_-saturated 0.5 M KHCO_3_ electrolyte: (a) BP, (b) In/BP-53 and (c) Cu/BP-53.


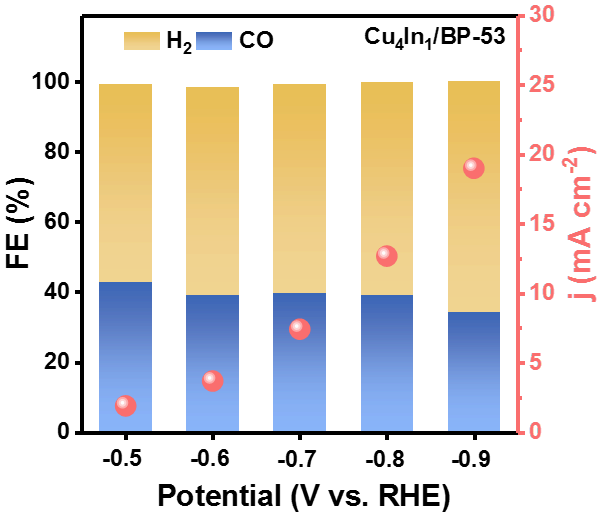


**Figure S18.** CO_2_RR performances of Cu_4_In_1_/BP-53 sample under different potentials.


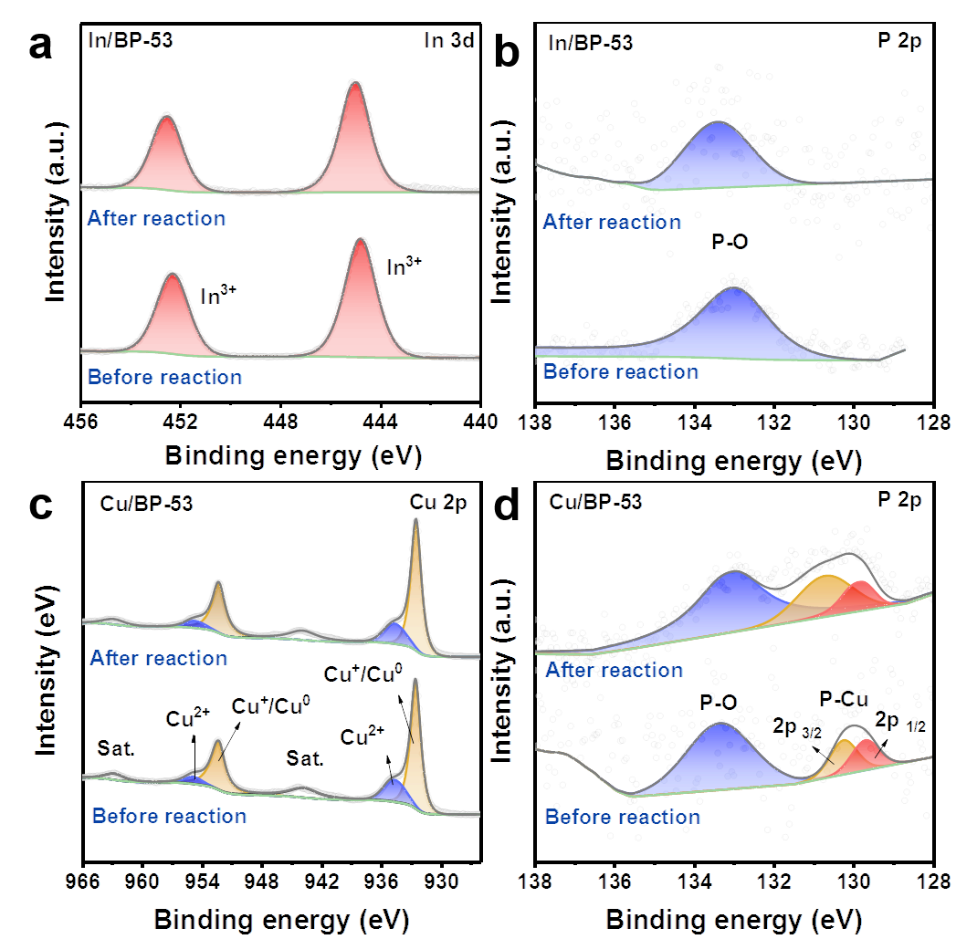


**Figure S19.** The XPS spectra of In/BP-53 before and after CO_2_RR reaction: (a) In 3d, (b) P 2p. The XPS spectra of Cu/BP-53 before and after CO_2_RR reaction: (c) Cu 2p, (d) P 2p.


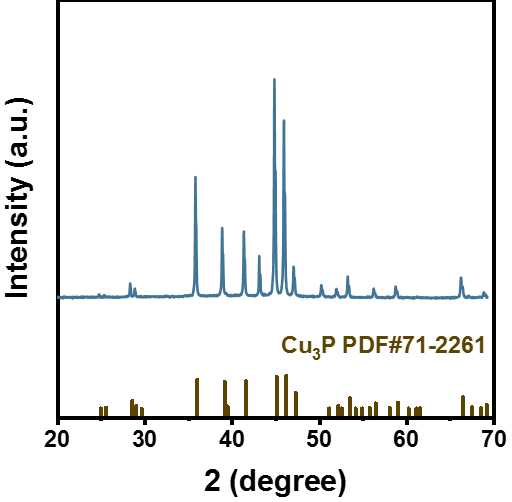


**Figure S20.** XRD patterns of Cu_3_P sample.


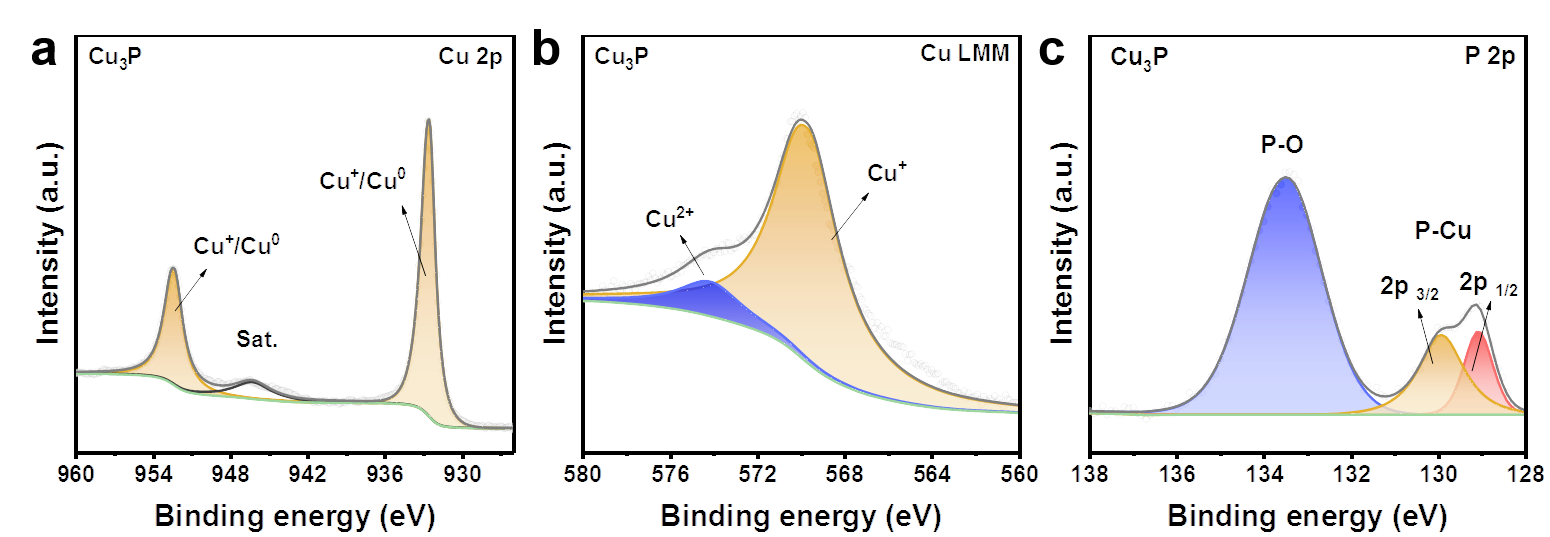
**Figure S21.** XPS spectra of Cu_4_In_1_: (a) Cu 2p, (b) Cu LMM and (c) P 2p.


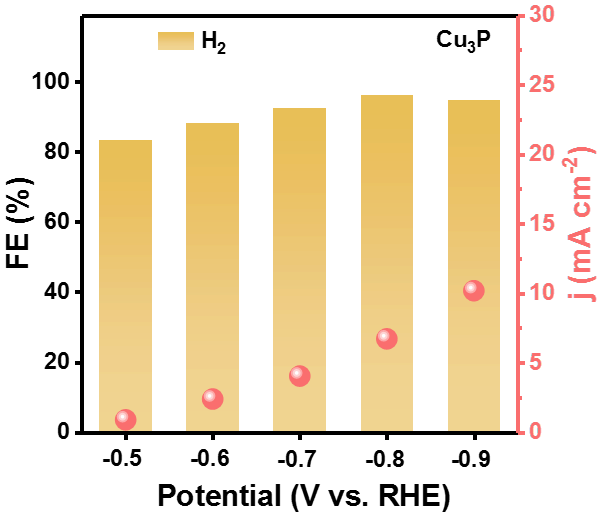


**Figure S22.** CO_2_RR performances of Cu_3_P sample under different potentials.


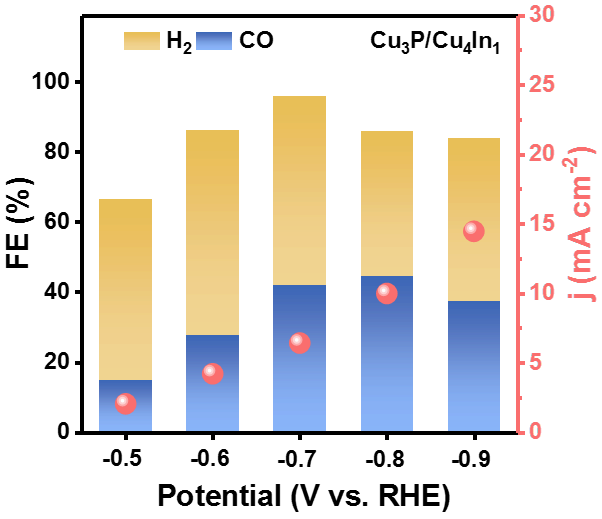


**Figure S23.** CO_2_RR performances of Cu_3_P/Cu_4_In_1_ composite sample under different potentials.


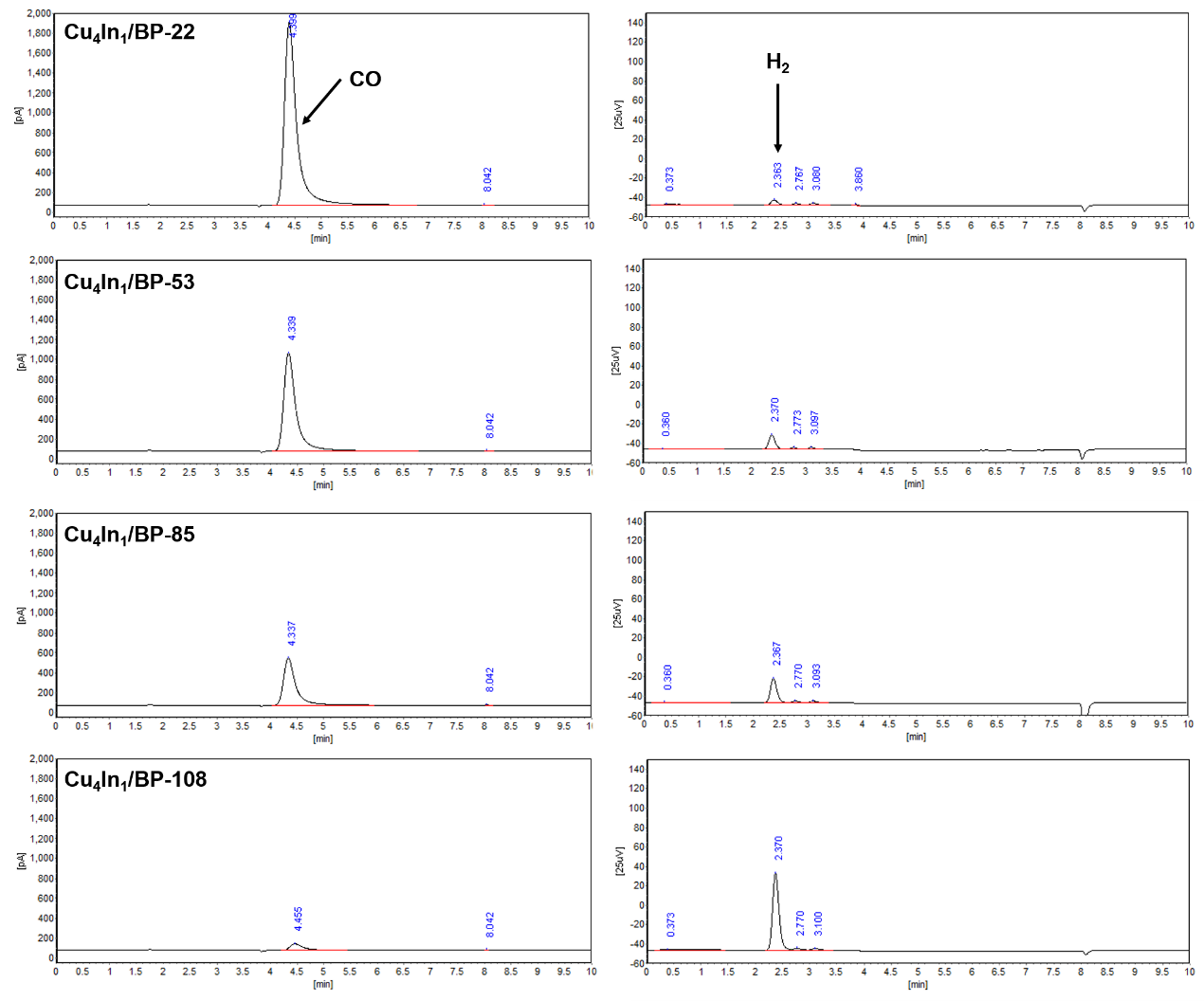


**Figure S24.** Representative gas-chromatography data of gas products of Cu_4_In_1_/BP with different BP molar percentage.

**
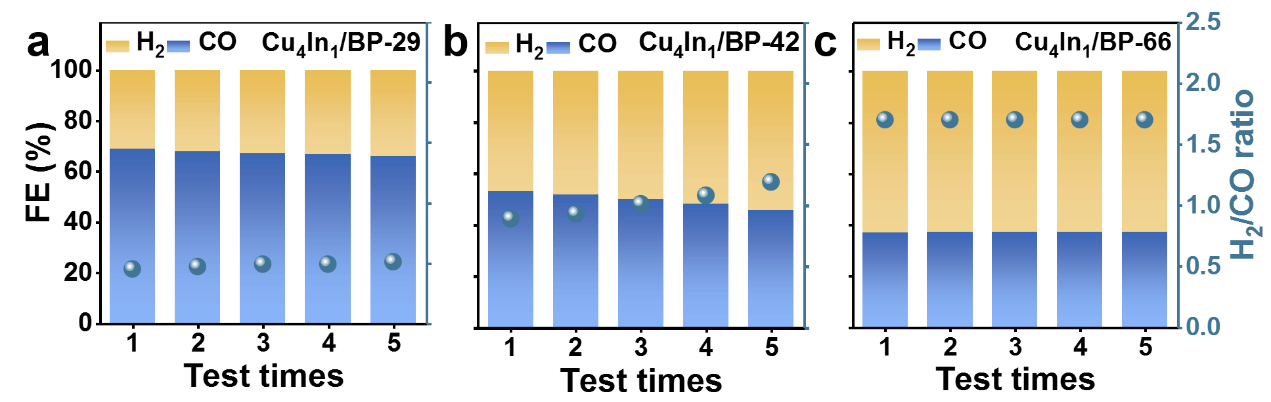
**

**Figure S25.** The faradic efficiencies and H_2_/CO ratio of Cu_4_In_1_/BP with specific BP molar percentage (-0.6 V vs. RHE, five tests): (a) molar percentage of 0.29, (b) molar percentage of 0.42, (c) molar percentage of 0.66.


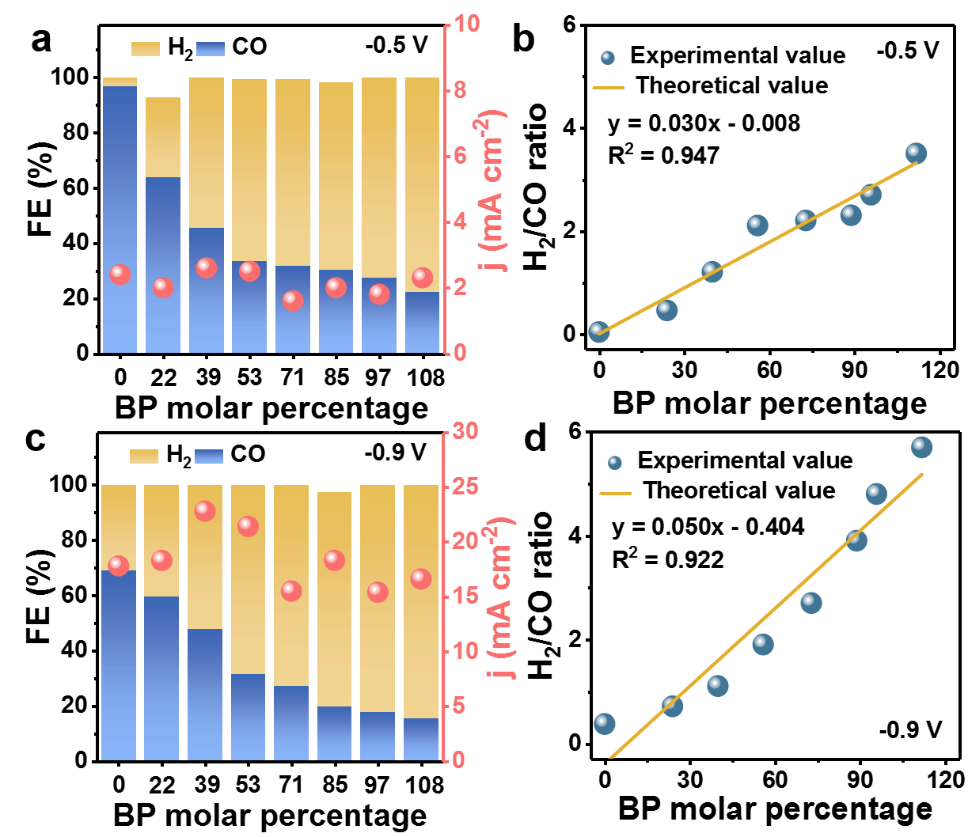


**Figure S26.** At -0.5 V vs. RHE, (a) the CO_2_RR performance of Cu_4_In_1_/BP with different BP molar percentage and (b) the fitted linear curve of BP molar percentage in Cu_4_In_1_/BP and H_2_/CO ratio. At -0.9 V vs. RHE, (c) the CO_2_RR performance of Cu_4_In_1_/BP with different BP molar percentage and (d) the fitted linear curve of BP molar percentage in Cu_4_In_1_/BP and H_2_/CO ratio.


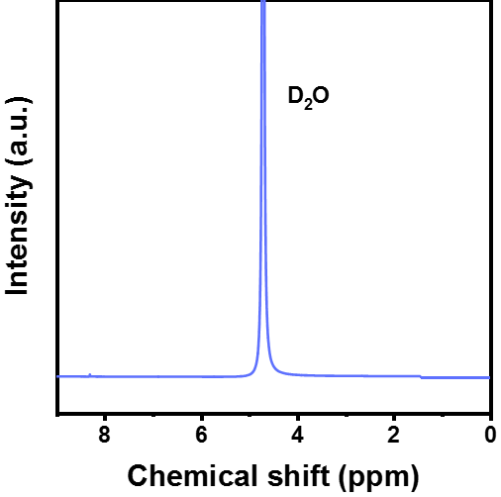


**Figure S27.** ^1^H NMR spectroscopy for liquid products of Cu_4_In_1_/BP-53 after CO_2_RR.


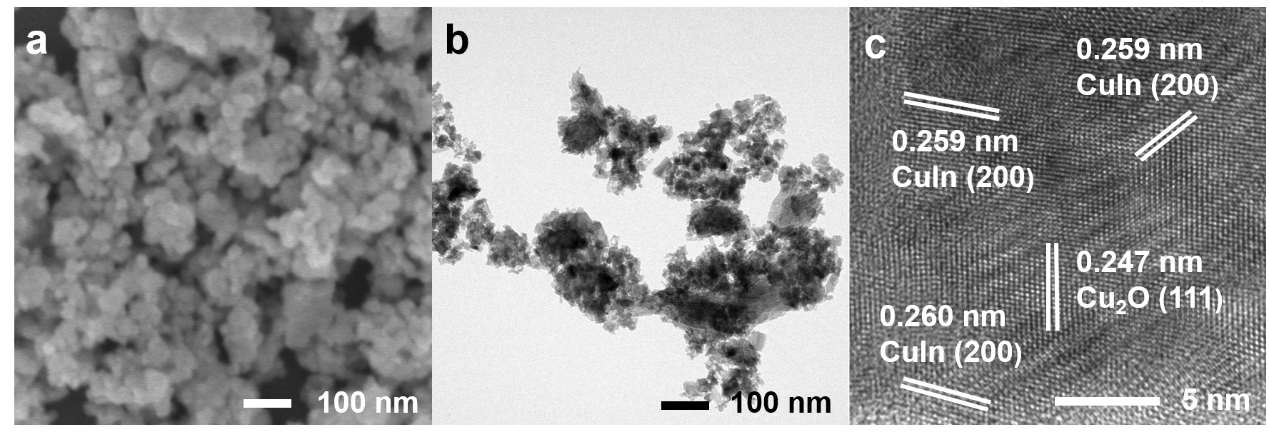


**Figure S28.** (a) SEM, (b) TEM and (c) HRTEM images of Cu_4_In_1_/BP-53 after CO_2_RR.


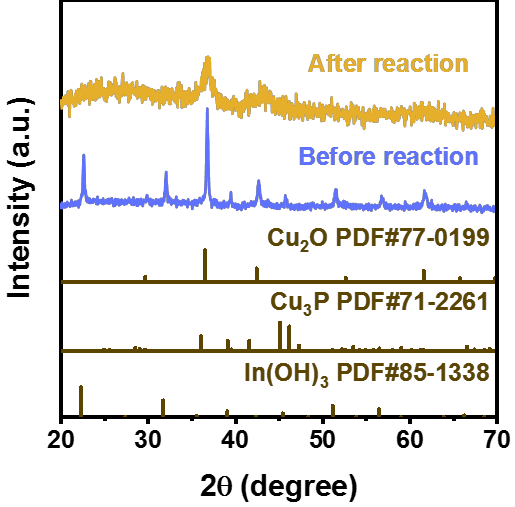


**Figure S29.** XRD patterns of Cu_4_In_1_/BP-53 before and after CO_2_RR.


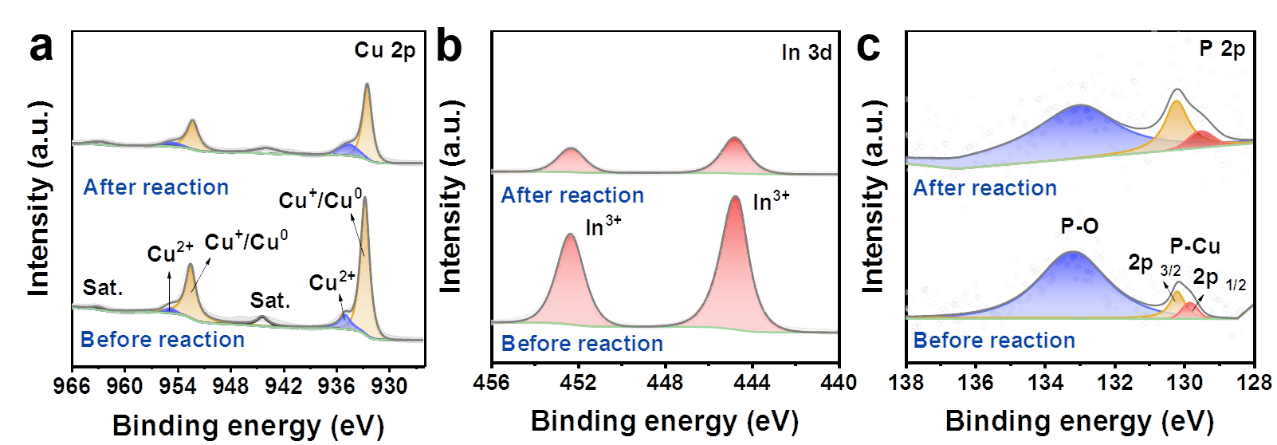


**Figure S30.** XPS spectra of Cu_4_In_1_/BP-53 before and after CO_2_RR: (a) Cu 2p, (b) In 3d and (c) P 2p.


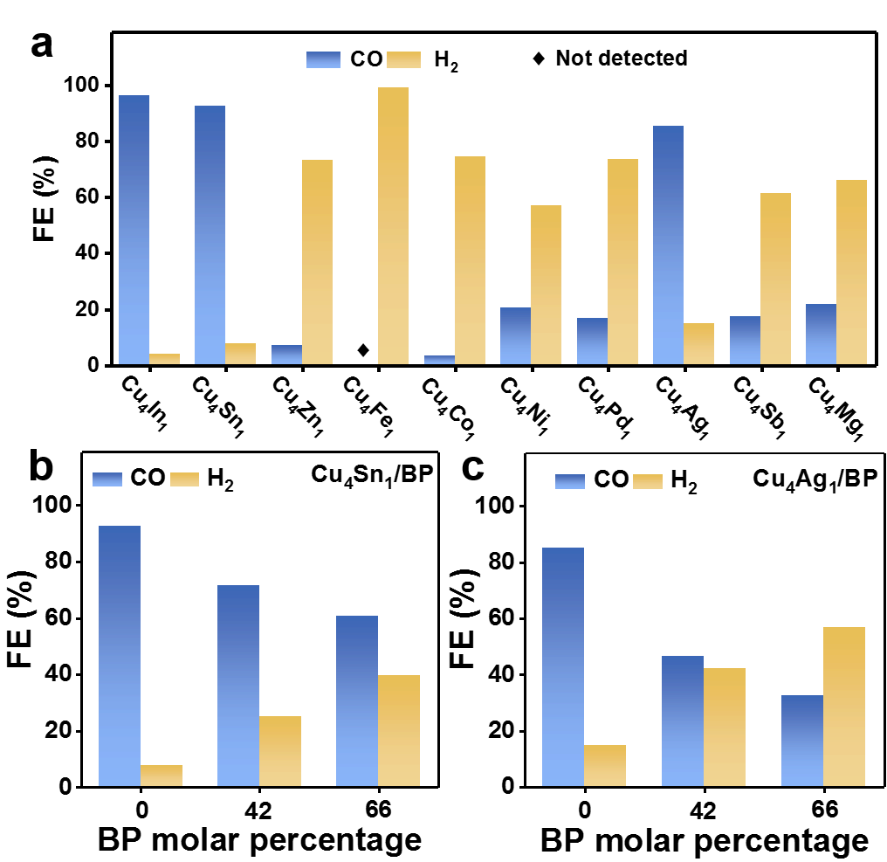


**Figure S31.** (a) At -0.6 V vs. RHE, the faraday efficiencies of CO and H_2_ over Cu_4_M_1_ catalysts. At -0.6 V vs. RHE, the faraday efficiencies of CO and H_2_ production over Cu_4_Sn_1_/BP (b) and Cu_4_Ag_1_/BP (c) with different BP molar percentage.


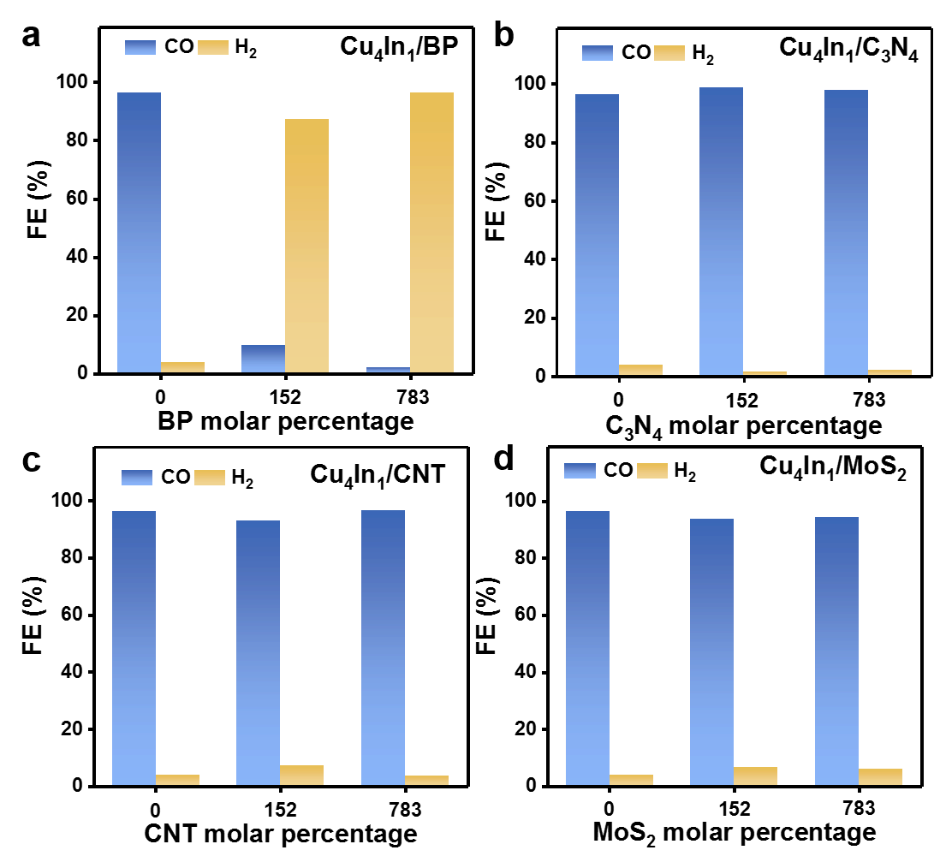


**Figure S32.** At -0.6 V vs. RHE, the faraday efficiency of CO and H_2_ production after four different materials are coupled with Cu_4_In_1_: (a) Cu_4_In_1_/BP, (b) Cu_4_In_1_/C_3_N_4_, (c) Cu_4_In_1_/CNT, (d) Cu_4_In_1_/MoS_2_.


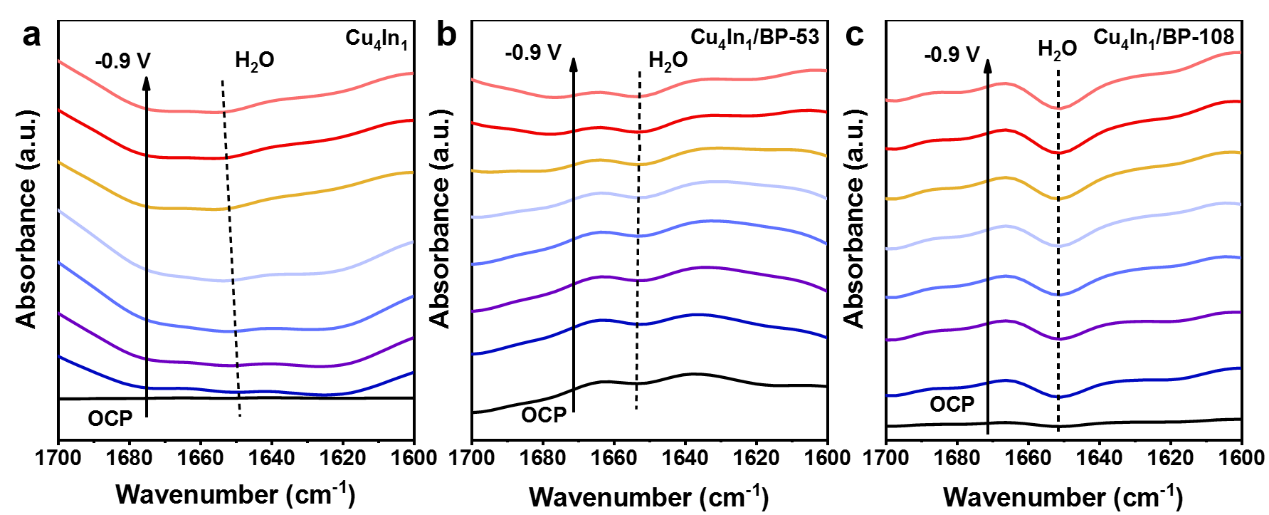


**Figure S33.** *In situ* ATR-FTIR spectra of (a) Cu_4_In_1_, (b) Cu_4_In_1_/BP-53, and (c) Cu_4_In_1_/BP-108 during CO_2_RR in CO_2_-saturated 0.5 M KHCO_3_ electrolyte.


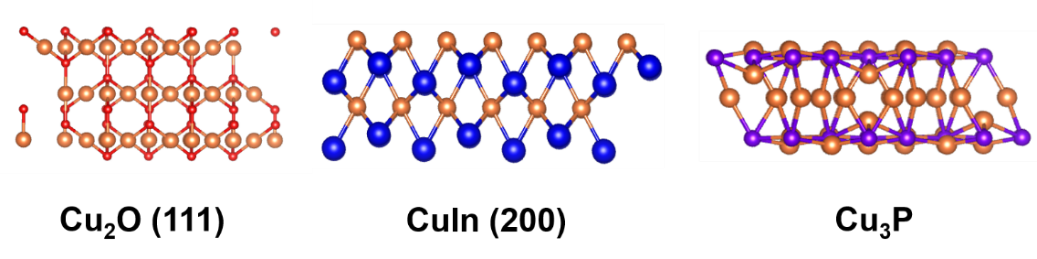


**Figure S34.** The crystal structures of the Cu_2_O (111), CuIn (200), and Cu_3_P models.


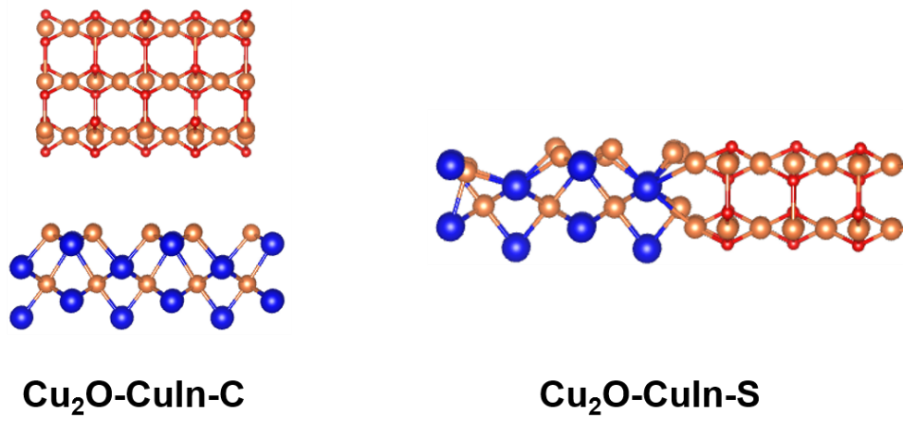


**Figure S35.** The crystal structures of the Cu_2_O-CuIn-C and Cu_2_O-CuIn-S models.


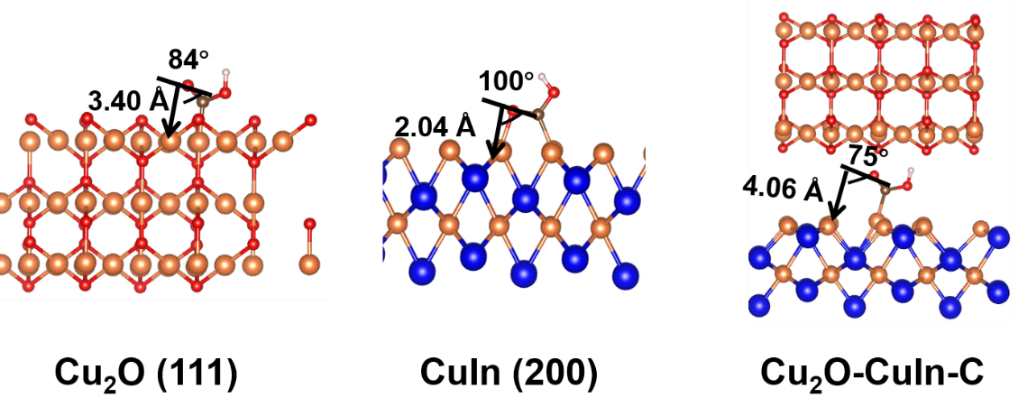


**Figure S36.** Optimized structure and induced charge for *COOH on Cu_2_O (111), CuIn (200) and Cu_2_O-CuIn-C.


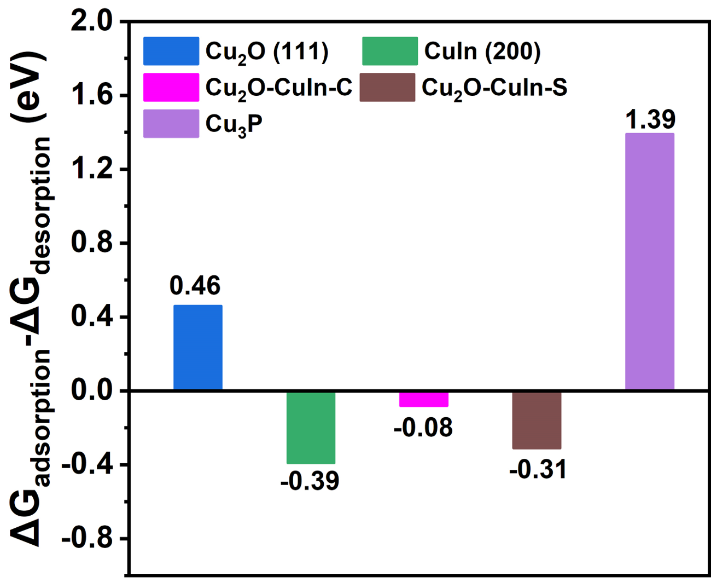


**Figure S37.** The free energy difference between *COOH adsorption and CO desorption on Cu_2_O (111), CuIn (200), Cu_3_P, Cu_2_O-CuIn-C and Cu_2_O-CuIn-S surface.

**Table S1.** Precursors used in 10 different Cu_4_M_1_ catalysts.

| **Sample** | **Cu precursor** | **Another metal precursor** |
| --- | --- | --- |
| **Cu_4_In_1_** | Cu(NO_3_)_2_·3H_2_O | In(NO_3_)_3_·xH_2_O |
| **Cu_4_Sn_1_** | CuCl_2_·2H_2_O | SnCl_4_ |
| **Cu_4_Zn_1_** | Cu(NO_3_)_2_·3H_2_O | Zn(NO_3_)_2_·6H_2_O |
| **Cu_4_Fe_1_** | CuCl_2_·2H_2_O | FeCl_3_ |
| **Cu_4_Co_1_** | CuCl_2_·2H_2_O | CoCl_2_ |
| **Cu_4_Ni_1_** | Cu(NO_3_)_2_·3H_2_O | Ni(NO_3_)_2_·6H_2_O |
| **Cu_4_Pd_1_** | Cu(CH_3_COO)_2_·H_2_O | Pd(CH_3_COO)_2_ |
| **Cu_4_Ag_1_** | Cu(NO_3_)_2_·3H_2_O | AgNO_3_ |
| **Cu_4_Sb_1_** | Cu(CH_3_COO)_2_·H_2_O | Sb(CH_3_COO)_3_ |
| **Cu_4_Mg_1_** | CuCl_2_·2H_2_O | MgCl_2_ |

**Table S2.** The molar ratios of Cu and In in Cu_4_In_1_/BP-53 and Cu_4_In_1_ samples

| **Samples** | **Cu:In (STEM-EDS)** | **Cu:In (ICP-OES)** |
| --- | --- | --- |
| Cu_4_In_1_/BP-53 | 3.3:1 | 3.2:1 |
| Cu_4_In_1_ | 2.7:1 | 3.0:1 |

**Table S3.** The formation energy of different catalysts.

| **Samples** | **E_b_ (eV)** |
| --- | --- |
| Cu_2_O (111) | -1.22 |
| CuIn (200) | -0.71 |
| Cu_3_P | -1.44 |
| Cu_2_O-CuIn-C | -0.96 |
| Cu_2_O-CuIn-S | -0.66 |

**Reference**

1. G. Kresse, J. Furthmüller, Efficiency of ab-initio total energy calculations for metals and semiconductors using a plane-wave basis set. *Comput. Mater. Sci.* **1996**, 6, 15.
2. G. Kresse, J. Hafner, Ab initio molecular dynamics for open-shell transition metals. *Phys. Rev. B.* **1993**, 48, 13115.
3. G. Kresse, D. Joubert, From Ultrasoft Pseudopotentials to the Projector Augmented-Wave Method. *Phys. Rev. B.* **1999**, 59, 1758.
4. P. Blöchl, Projector Augmented-Wave Method. *Phys. Rev. B.* **1994**, 50, 17953.
5. H. Monkhorst, J. Pack, Special Points for Brillonin-Zone Integrations. *Phys. Rev. B.* **1976**, 13, 5188.
6. J. Nørskov, J. Rossmeisl, A. Logadottir, L. Lindqvist, Origin of the Overpotential for Oxygen Reduction at a Fuel-Cell Cathode. *J. Phys. Chem. B.* **2004**, 108, 17886.
7. X. Liang, X. Ren, M. Guo, Y. Li, W. Xiong, W. Guan, L. Gao, A. Liu, CO_2_ Electroreduction by AuCu Bimetallic Clusters: A First Principles Study. *Int J Energy Res.* **2021**, 45, 18684.
8. L. Zhang, Y. Meng, J. Yang, H. Shen, C. Yang, B. Xie, S. Xia, Theoretical study on dry reforming of methane catalyzed by Cu_12_M (M = Cu, Fe, Co, Ni) core-shell bimetallic clusters. *Fuel* **2021**, 303, 121263.
